# Near Surface Swimming of *Salmonella* Typhimurium Explains Target-Site Selection and Cooperative Invasion

**DOI:** 10.1371/journal.ppat.1002810

**Published:** 2012-07-26

**Authors:** Benjamin Misselwitz, Naomi Barrett, Saskia Kreibich, Pascale Vonaesch, Daniel Andritschke, Samuel Rout, Kerstin Weidner, Milos Sormaz, Pascal Songhet, Peter Horvath, Mamta Chabria, Viola Vogel, Doris M. Spori, Patrick Jenny, Wolf-Dietrich Hardt

**Affiliations:** 1 Institute of Microbiology, D-BIOL, ETH Zürich, Zurich, Switzerland; 2 Department of Materials, ETH Zurich, Zürich, Switzerland; 3 Light Microscopy Centre, ETH Zurich, Zürich, Switzerland; 4 Department of Health Sciences and Technology, ETH Zurich, Zürich, Switzerland; 5 Institute for Fluid Dynamics, ETH Zurich, Zürich, Switzerland; Yale University School of Medicine, United States of America

## Abstract

Targeting of permissive entry sites is crucial for bacterial infection. The targeting mechanisms are incompletely understood. We have analyzed target-site selection by *S*. Typhimurium. This enteropathogenic bacterium employs adhesins (e.g. *fim*) and the type III secretion system 1 (TTSS-1) for host cell binding, the triggering of ruffles and invasion. Typically, *S*. Typhimurium invasion is focused on a subset of cells and multiple bacteria invade via the same ruffle. It has remained unclear how this is achieved. We have studied target-site selection in tissue culture by time lapse microscopy, movement pattern analysis and modeling. Flagellar motility (but not chemotaxis) was required for reaching the host cell surface in vitro. Subsequently, physical forces trapped the pathogen for ∼1.5–3 s in “near surface swimming”. This increased the local pathogen density and facilitated “scanning” of the host surface topology. We observed transient TTSS-1 and *fim*-independent “stopping” and irreversible TTSS-1-mediated docking, in particular at sites of prominent topology, i.e. the base of rounded-up cells and membrane ruffles. Our data indicate that target site selection and the cooperative infection of membrane ruffles are attributable to near surface swimming. This mechanism might be of general importance for understanding infection by flagellated bacteria.

## Author Summary

The animal body is protected by physical, chemical and immunological barriers. Identification of “promising” target sites is therefore of importance for any pathogen. This crucial step of the infection is still poorly understood. Here, we have studied target site selection by the flagellated Gram-negative gut pathogen *Salmonella* Typhimurium. Using a well-established tissue culture model system, we found that flagella-driven motility forces the bacterium into a “near surface swimming” mode which facilitates “scanning” of the host cell surface. The near surface swimming was found to target the pathogen towards sites with particular topological features, i.e., rounded cells and membrane ruffles. This explains how *S*. Typhimurium “identifies” particular target sites and infects membrane ruffles in a cooperative manner. Interestingly, the near surface swimming is attributable to generic physical principles acting on moving particles. Therefore, our findings might be of general importance for the infection by motile pathogens.

## Introduction

*Salmonella enterica* subspecies 1 serovar Typhimurium (referred to as *S*. Typhimurium in this study) is a common food-borne pathogen. Central to the pathogenesis of *S*. Typhimurium is its ability to invade intestinal cells, namely M-cells, epithelial cells and possibly dendritic cells [1], [2], [3]. Normally, only a small fraction of the mucosal cells are being invaded [4], [5], [6], [7]. The mechanisms focusing *S*. Typhimurium invasion to particular sites are not completely understood.

Host-cell invasion by *S*. Typhimurium is the result of a multistep process. This includes: i) 3-dimensional movement in the gut lumen (motility, chemotaxis, diffusion); ii) transient interactions with the mucosal surface and particulate matter within the gut lumen; iii) reversible binding via adhesins like type 1 fimbriae (*fimH*, [8], [9]); iv) irreversible “docking” mediated via type III secretion system 1 (TTSS-1; [8], [9]). This step commits wild type *S*. Typhimurium to invasion. v)secretion of bacterial virulence factors, so called effectors, via TTSS-1 into the host cytosol; key effectors include SopE, SopE2, SopB and SipA.; vi) manipulation of the host cell by *S*. Typhimurium effectors leading to the emergence of prominent membrane ruffles in epithelial- and M-cells [10]; vii) host cell invasion, which often features the simultaneous entry of several bacteria through the same ruffle. While many steps have been studied in detail before, those steps targeting the pathogen to a particular site (i.e. steps ii and iii) have remained enigmatic. Thus, it is still unclear whether *S*. Typhimurium actively “selects” target sites and which mechanism would enable such a preference.

We speculated that motility might affect target site selection. Like many other pathogens, *S.* Typhimurium employs flagella to orient and move in 3D space [11], [12].This has multiple well documented effects on the pathogen-host interaction. The coupling to chemo-sensing systems allows swimming towards nutrient sources (“chemotaxis”; [13], [14]). Motility is important for the invasion of tissue-culture cells and in the induction of gut inflammation by *Salmonella* spp. (in vitro: [15], [16], [17], [18], [19]; animal model: [20], [21], [22], [23], [24]). Furthermore, the flagella might mediate adhesion [25] or elicit host-cellular signaling responses. It remained unclear whether flagella may also serve additional tasks, i.e. in target site selection.

Flagellar rotation propels the bacterium with a velocity of at least 25–55 µm/s(“motility” [12], [26]).If encountering a host cell, *S*. Typhimurium is generally assumed to either be “deflected” back into the medium or to initiate a productive infection. However, so far, this step of the infection process has not been studied in detail.

**Figure 1:**
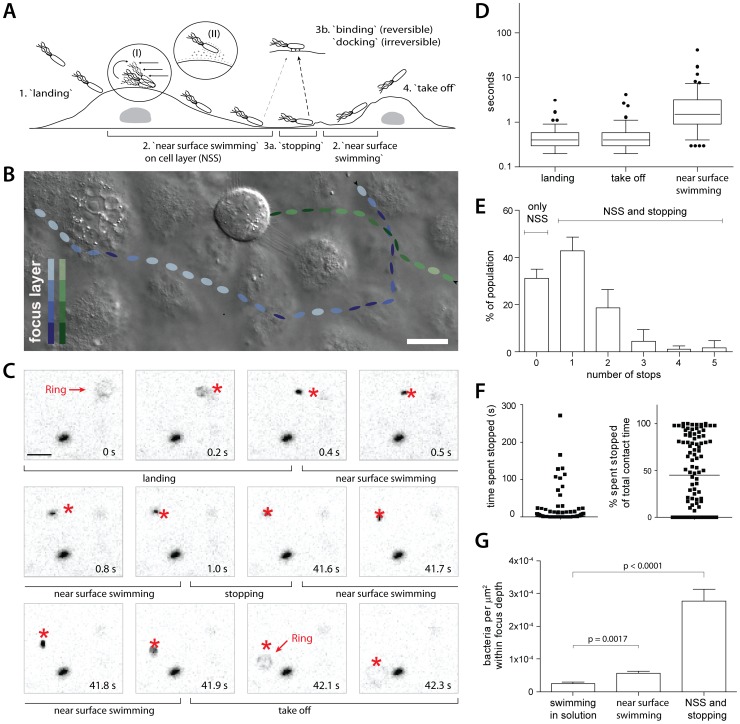
Near surface swimming of *S*. Typhimurium on cellular surfaces. (A) Scheme describing the four stages of *S*. Typhimurium movement observed at cellular surfaces. Inserts (I) and (II) indicate two possible mechanisms for trapping *S*. Tymphimurium in proximity to the surface: hydrodynamic entrapment (I) and DLVO interactions (II), respectively. A “stop” on the surface can be due to an obstruction hindering the path of the bacterium (3a) or reversible binding/irreversible docking (3b), see text for details. (B) Snapshot of a movie acquired using DIC imaging. HeLa cells were infected with *S*.Tm^Δ4^ and the interactions of bacteria with cells were followed in real time. The tracks of 2 representative *S*.Tm^Δ4^bacteria are indicated, their estimated positions in the Z- layer while moving along the cellular surface are indicated by the shade of color. The bacterium indicated in green encounters a mitotic cell and stops until the end of the movie while the bacterium indicated in blue crosses and leaves the field of view. Compare supplementary Videos S1 and S2. Scale bar: 18 µm. (C) HeLa cells were infected with *S*.Tm^Δ4^(pGFP) and 5-minute fluorescence microscopy movies were acquired. Representative frames illustrating key stages of *S*. Typhimurium NSS and our quantification strategy are depicted (see text as well as Materials and Methods for details). In each frame, the star indicates the position of the corresponding bacterium in the previous frame. Note the fluorescent ring in time points 0 s and 42.1 s, indicating the start (“landing”) and end (“take off”) of the contact with the host cell. (D) Quantitative analysis of the different stages of NSS (see A and C) for *S*.Tm^Δ4^(pGFP). 5 independent experiments were analyzed (n = 122 bacteria). The box plot represents the median, interquartile range, the 5%–95% range as well as outliers. (E) Quantification of the fraction of *S*.Tm^Δ4^(pGFP) making no stops (“NSS only”), or the fraction making the indicated number of stops on the cell surface during NSS (“NSS and stopping”; further analysis of the experiment in D). Error bars:standard deviation. (F) Quantitative analysis of the time a bacterium spends stopped at the surface. The data is plotted either in seconds (left panel) or as a fraction of the total contact time of the respective bacterium (right panel; further analysis of a subset of the experiment in D). (G) HeLa cells were infected with *S*.Tm^Δ4^(pGFP) and 5-minute fluorescence movies were acquired focusing either >100 µm above the cells (“swimming in solution”) or on the cell layer. For 40 time points from 2 independent experiments the number of *S*.Tm^Δ4^(pGFP) in a field of view was quantified. Either the whole population (“NSS and stopping”) or only moving bacteria (“NSS”, immotile bacteria excluded) were counted. **: p<0.01; ***: p<0.0001.

In contrast, the interaction of motile *E. coli* strains and solid surfaces has been extensively studied. On solid surfaces, *E. coli* slides in large circles, remaining in contact with the surface for extended time periods, a phenomenon called near surface swimming (NSS). Two mechanisms explaining this ability of *E*. *coli* to swim along solid surfaces have been proposed (Fig. 1A, inserts I and II). According to the hydrodynamic entrapment theory [27], [28], the bacteria experience extensive drag stress at the part of their body close to the surface, causing a “forward” rotation. This rotates the rod-shaped bacterium into an “upright” position. The upright position in turn increases the drag resistance against the fluid, resulting in an opposing rotational force. Ultimately, these two forces are in equilibrium, keeping the bacterial rod at a constant angle towards the surface, thus entrapping the organism in a tilted swimming position. The alternative DLVO model (Derjaguin, Landau, Verwey, and Overbeek; for a review, see [29]) explains NSS via electrostatic and van der Waals forces. Nevertheless, both models predict that motile bacteria encountering a solid surface would be “trapped” at the surface and perform a NSS motion. It remains unclear whether NSS may also occur on cellular surfaces and whether this might affect target site selection.

Here, we studied target site selection by *S*. Typhimurium. The initial stages of *S.* Typhimurium-interaction with cellular or artificial surfaces were analyzed in real time. In this “pre-docking” phase of the infection, bacterial motility was of key importance. It led to characteristic near surface swimming patterns on host-cell surfaces and targeting to sites with a prominent surface topology. Our data suggest a model, in which physical forces emanating from the flagella-driven motility facilitate near-surface swimming and explain the pathogen's target preference during infection. We are discussing possible implications for the disease and for infections by other flagellated pathogens.

## Results

### Time-lapse microscopy reveals swimming of wild type *S*. Typhimurium near cellular surfaces

To study the initial interactions of *S*. Typhimurium with cellular surfaces, we employed time-lapse microscopy (supplementary Videos S1 and S2). HeLa cells, a commonly used tissue-culture model for studying *S.* Typhimurium invasion, were infected with *S*.Tm^Δ4^ (SL1344 *sopEE2B sipA*; Table 1). *S*.Tm^Δ4^behaves like *S*.Tm^wt^ in all aspects of the early host-cell interaction (steps i to v), but cannot trigger membrane ruffling or invasion as it lacks the key effector proteins SopE, SopE2, SopB and SipA [8], [9], [30].Therefore, *S*.Tm^Δ4^ allowed us to focus on the initial surface interactions and docking (Fig. 1A).

**Table 1:** Strains and plasmids used.

Strain/Plasmid	Genotype	Alternative name	Reference
*S.*Tm^wt^		SL1344, SB300	[53]
*S.*Tm^Δ4^	Δ*sipAsopBEE2*	M566	[54]
*S.*Tm^-T1^	Δ*invG*	SB161	[55]
*S.*Tm^-T1 -Fi^	Δ*invG, fimD::pGP704*	M1915	[9]
*S*.Tm^SopE^	Δ*sopB*, *sipA::aphT*, *sopE2::tet*	M701	[5]
*S.*Tm^Δ4cheY^	Δ*sipAsopBEE2, cheY::Tn10*	M1918	this study
*S.*Tm^fliGHI^	*fliGHI::Tn10*	M913	[22]
*S.*Tm^Δ4 fliGHI^	Δ*sipAsopBEE2*, *fliGH::Tn10*	M1921	this study
*S.*Tm^Δ4motAB^	Δ*sipAsopBEE2, motAB::CmR*	M1927	this study
*S.*Tm^Δ4flgK^	Δ*sipASopBEE2, flgK::CmR*	M1924	this study
		M2424	[35]
*S.*Tm^-T1 fliGHI^	Δ*invG, fliGH::Tn10*	M1920	this study
*S.*Tm^-T1motAB^	Δ*invG*, *motAB::CmR*	M1926	this study
*S.*Tm^-T1flgK^	Δ*invG*, *flgK::CmR*	M1923	this study
*S.*Tm^-T1 -Fi fliGHI^	Δ*invG, fimD::pGP704,fliGHI::Tn10*	M1919	this study
*S.*Tm^-T1 -Fi motAB^	Δ*invG, fimD::pGP704, motAB::CmR*	M1925	this study
*S.*Tm^-T1 -Fi flgK^	Δ*invG, fimD::pGP704, flgK::CmR*	M1922	this study
*E.coli* Nissle	motile non-pathogenic		[56]
pGFP used in all time lapse experiments	constitutive *gfp*-expression	pM965	[22]
pM975	*gfp*-expression in intracellular *S.* Typhimurium		[4]
pGFP used in all other experiments	constitutive *gfp*-expression	pCJLA-GFP	[57]
pM2112	constitutive *rfp*-expression		this study

First, the initial surface interactions of *S*.Tm^Δ4^were analyzed using time-lapse differential-interference contrast (DIC) microscopy (Fig. 1B).Strikingly, *S*.Tm^Δ4^was swimming along the cellular surface for extended time periods (supplementary Videos S1 and S2). Our subsequent frame-by-frame analysis of the time-lapse videos identified several “stages” of this interaction:

- “landing”: bacteria leaving the bulk medium to come into close proximity to the host;
- “near surface swimming” (NSS), defined as a continuous movement along the surface of the host cell;
- “stopping”:NSS is discontinued and bacteria remain at one particular spot on/near the host cell surface for some time(0.3 s≤stopping time≤remaining length of movie);
- “take off”: bacteria leaving the cell surface (Fig. 1A,B; supplementary Videos S1, S2).

In most cases, bacteria went through all stages of interaction before taking off again. Please note that some bacteria stopped until the end of the time-lapse movie. In this experiment, we could not distinguish whether these bacteria were “docking” (i.e. bacteria that bound irreversibly, e.g. via the TTSS-1 apparatus [8], [9], [30]), or “stopping” (bacteria transiently stopping but continuing NSS or taking off after the end of the movie).Stopping did not happen randomly but occurred frequently at “obstacles” encountered during NSS. In particular, *S.* Typhimurium stopped and docked at cells with a round morphology(i.e. a mitotic cell; see supplementary Video S1). This provided a first indication that a transient stop may “preselect” specific sites for subsequent docking of *S*. Typhimurium. This would be in line with the preferential docking of *S*. Typhimurium onto mitotic cells observed in earlier studies ([31], [32], see below).

For a quantification of these initial bacteria surface interactions we used *S*.Tm^Δ4^harboring a plasmid conferring constitutive *gfp* expression (pGFP;Table 1) and time lapse fluorescence microscopy. This allowed precise quantification of all stages of the bacteria surface interaction including landing and take-off, since the fluorescent bacteria moving out of the focus layer appear as “rings” in the movie (see T0s in Fig. 1C). Hence, the “landing” stage was defined as the time between the first detection of a “ring” (followed by a continuous downward movement) and the change of the direction and speed typically observed when NSS started(T0–0.4 s in Fig. 1C). In analogy, the “take off”-stage describes the time between the end of stopping or NSS and the disappearance of the “ring” (T41.9–42.3 s in Fig. 1C). Additionally, we tracked the time spent stopped or engaged in NSS. Take-off and landing occurred within <3.1 and <4.2 s, respectively (median 0.4 s for both; Fig. 1D), while the time engaged in NSS was significantly longer (median 1.5 s; range 0.3–41.5 s; Fig. 1D). Overall, the bacteria covered significant distances swimming along the host cellular surface(6.7–325 µm; see also below).Furthermore, we observed a variety of “behaviors” with respect to stopping.33% of all imaged *S*.Tm^Δ4^(pGFP) bacteria did never stop, while 67% made one or more stops (“NSS and stop”; Fig. 1E). Some bacteria stopped up to 5 times on the cell surface and some *S*.Tm^Δ4^remained stopped (or docked)until the end of the imaging experiment, with the longest observed stop lasting 280 s (Fig. 1F).

Overall, the bacterial density at the cellular surface was significantly increased compared to the overlaying media (Fig. 1G).Accumulation of bacteria at the cellular surfaces was not attributable to gravity, as indicated by a comparison between motile (affected by gravity and NSS) and non-motile bacteria (affected only by gravity; suppl. Fig. S1). Much rather, flagella-driven NSS and ensuing stops accounted for the increased local *S*. Typhimurium density at the host cell surface. This suggested that the prolonged contact time might contribute to the target site selection by *S*. Typhimurium.

### Fim-adhesins and TTSS-1 do not affect the initial surface interactions, i.e. landing, NSS, stopping or take-off

In order to determine the role of bacterial adhesins in the initial phases of the surface interaction, we analyzed two *S.* Typhimurium mutants, *S*.Tm^-T1^ (SL1344 *invG*) and *S*.Tm^-T1-Fi^ (SL1344 *invG fimD*; Tab. 1). These mutants lack one or two surface structures, namely type 1 fimbriae and the TTSS-1 apparatus, which are known to mediate reversible binding and irreversible docking of *S*. Typhimurium to HeLa and other host cells [8], [9], [30]. However, a role of these adhesins for near surface swimming or stopping had not been addressed.

**Figure 2 ppat-1002810-g002:**
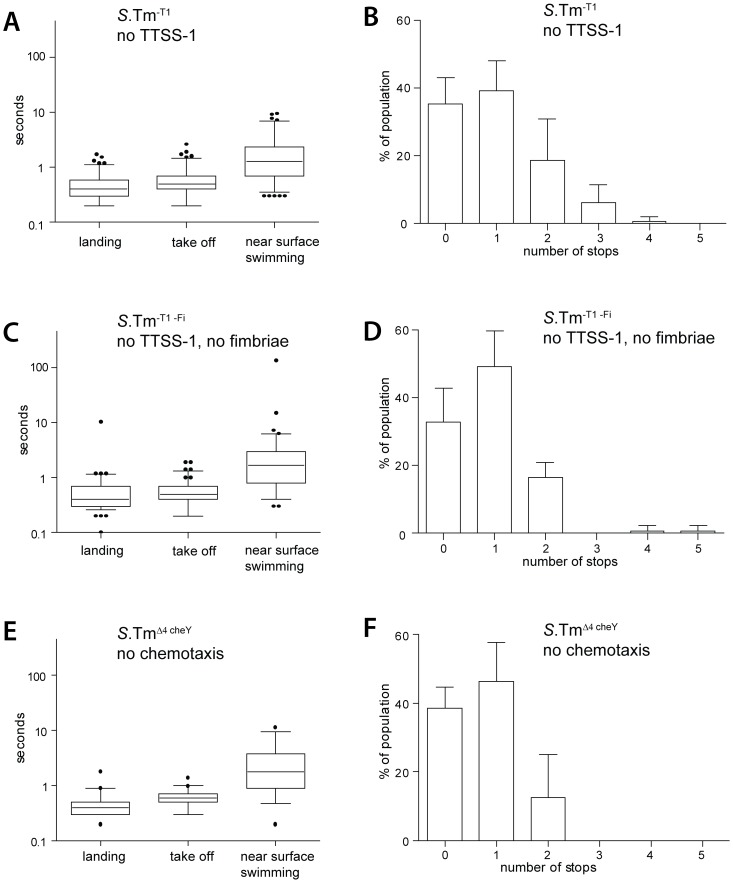
*S*. Typhimurium NSS does neither require adhesins nor chemotaxis. Quantitiative analysis of the initial host-cell interaction by*S*.Tm^-T1^(pGFP), *S*.Tm^-T1-Fi^(pGFP) and *S*.Tm^ΔcheY^(pGFP), lacking either the TTSS-1 apparatus,TTSS-1 and type 1 fimbriae or the four effectors SopE, SopE2, SopB and SipA as well as the protein CheY, essential for chemotaxis. (A), (C), (E)Different stages of NSS were quantified as described for Fig. 1D.The following numbers of bacteria were analyzed: *S*.Tm^-T1^(pGFP), n = 114; *S*.Tm^-T1-Fi^(pGFP),n = 91;*S*.Tm^ΔcheY^(pGFP), n = 39.Time-lapse microscopy data was derived from 2 to5independent experiments. (B), (D), (F) Numbers of stops by an individual bacterium were quantified as described in Fig. 1E.

HeLa cells were infected with *S*.Tm^-T1^(pGFP) and *S*.Tm^-T1-Fi^(pGFP) and transient interactions were monitored by time lapse fluorescence microscopy as in Fig. 1. All analyzed parameters, including the number of stops, were indistinguishable from those of *S*.Tm^Δ4^(Fig. 2A–D; compare to Fig. 1). These results indicated that none of the initial surface interactions were affected by TTSS-1or by *fimD*(Fig. 2A,C). Strikingly, this also pertained to the transient stops and clearly distinguishes the initial surface interactions from later stages of the infection, i.e. reversible binding and docking. Stopping thus seems to be attributable to a different mechanism.

Nevertheless, as indicated by earlier data [8], [9], [30],some of the stops must result in reversible binding and docking. To estimate the relative frequency of stopping and of the docking events, the total number of stops observed on the cell layer ( = the number of potential docking events), during a 5 minute period was calculated: the total number of *S*.Tm^Δ4^bacteria landing on the cell surface during a 5 min period was multiplied by the number of stops using the values determined in as Fig. 1E.After the end of the5-minute real time imaging experiment, the cells were washed, fixed and stained for DNA (DAPI), actin (TRITC-phalloidin) and *S.* Typhimurium (anti-LPS antibody).This protocol removed all “stopped” bacteria, while “reversibly bound” and “docked” bacteria remained on the cells and were enumerated by fluorescence microscopy [8], [9], [30]. Comparing the results from both types of analysis revealed that no more than 1–2% of the total stops (as detected by time lapse microscopy) resulted in a docking event. Hence, transient stops are approx. 50- to 100-fold more frequent than docking events, at least in the 5-minuteinfection experiments that we have performed, here.

In conclusion, these data established that NSS requires neither TTSS-1nor type I fimbriae. The initial pathogen host cell interaction is thus clearly distinct from subsequent stages, i.e. reversible binding and docking.

### *S*. Typhimurium near surface swimming does not require chemotaxis

Bacterial flagellar movement is guided by chemotaxis. It had remained unclear, if NSS was dependent on chemotaxis or whether non-directed motility would suffice. To address this issue, HeLa cells were infected with *S*.Tm^Δ4 cheY^(pGFP) (SL1344 Δ*sipAsopBEE2 cheY*; Table 1). This isogenic mutant is a “straight-swimmer”, expresses wild type numbers of functional flagella, but cannot swim along chemical gradients. The initial surface interactions were monitored by time lapse microscopy as described in Figs. 1 and 2A–D. All analyzed parameters were indistinguishable from those of*S*.Tm^Δ4^, *S*.Tm^-T1^ or S.Tm^-T1-Fi^(compare Fig. 2E,F to Fig. 1D,E and Fig. 2A–D). Therefore, in our tissue culture assays non-directed motility was sufficient for facilitating the initial surface interactions.

### Are physical forces sufficient for explaining the initial surface interactions of *S*. Typhimurium?

**Figure 3:**
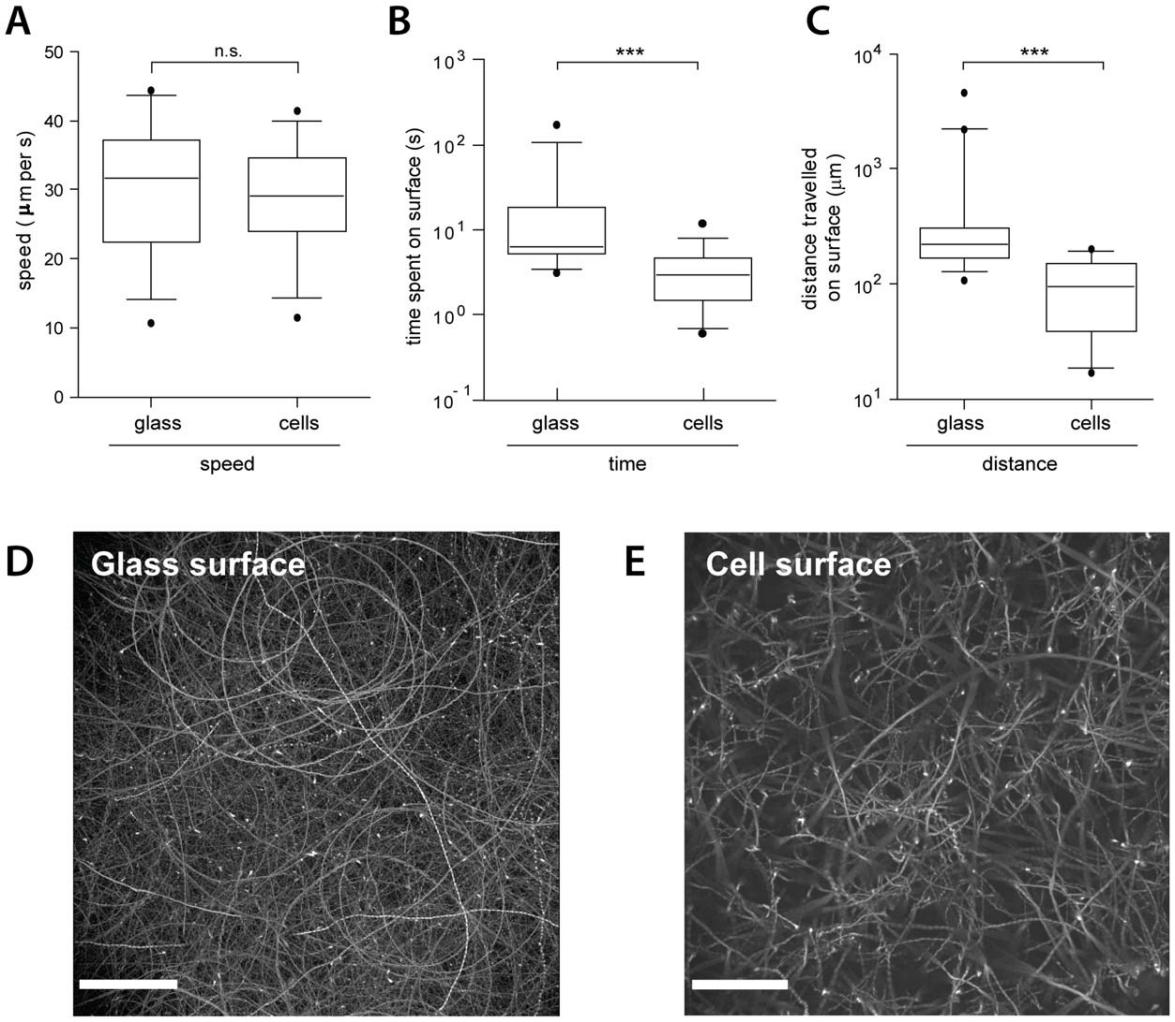
NSS of *S*. Typhimurium on cellular and artificial surfaces. Quantifications of time and distance of *S*.Tm^Δ4^(pGFP) swimming along the bottom of a glass dish, with or without seeded cells. (A–C) Comparison of the speed, the time spent in NSS, or the distance travelled on a cellular and a glass surface, respectively. The figure summarizes 17–22 bacteria per condition from 2 independent experiments. ***: p<0.0001. (D), (E) overlay of all frames (maximum intensity plot; Volocity software) from a 5-minute movie (10 frames/sec; Leica DMI-6000B, 20× air objective 0.7 NA, 2 fold optovar) in the GFP channel of *S*.Tm^Δ4^(pGFP) movement on a glass-or a cellular surface, respectively. Note the shorter and more linear tracks on the cellular surface. Scale bar: 57 µm.

Our data suggested that basic physical principles may explain the initial interactions of *S*. Typhimurium with host cellular surfaces. In this case, the movement patters on cellular and acellular surfaces should be quite similar. Therefore, we inoculated glass-bottom tissue culture dishes seeded with HeLa cells (or not) with *S*.Tm^Δ4^(pGFP). The bacterial surface interactions were analyzed by time-lapse fluorescence microscopy as described in Figs. 1 and 2. *S*.Tm^Δ4^(pGFP) performed NSS with equivalent speed on glass and on cellular surfaces (approx. 30 µm/s; Fig. 3A). However, the median duration of an episode of NSS(6.3 s on glass vs. 2.95 s on cells; Fig. 3B) and the median distance travelled during this time (221 µm on glass vs. 95 µm on cells; Fig. 3C) were slightly larger on glass than on the cellular surface. These observations were in line with our hypothesis that the initial surface interactions may be governed by equivalent physical principles.

Strikingly, the bacteria followed “right-handed” curved tracks on glass and, to a lesser extent, on cellular surfaces (Fig. 3D,E). It is thought that this curvature of the NSS tracks is attributable to the shear force between the flagella-mediated rotation of the bacterial body and the respective surface [28], [33].

So far, our data were in line with the hypothesis that general physical principles are responsible for NSS on cellular and on glass surfaces. In this case, other types of host cells or motile bacteria should yield similar results. Therefore, we have extended our analysis to MDCK cells, a commonly used polarized epithelial cell line, and *E. coli*Nissle which was transformed with the GFP expression plasmid (*E.coli*Nissle (pGFP); Suppl. Fig. S2). Both, *E.coli*Nissle (pGFP) and *S*.Tm^Δ4^ (pGFP) engaged in NSS and displayed similar movement patterns on MDCK, on HeLa cells and on glass surfaces (Fig. 3D,E and suppl. Fig. S2A–D). Nevertheless, the shape of the NSS-tracks may differ slightly. This might be attributable to different morphological features displayed by the different types of surface and represents an interesting topic for future research.

In conclusion, these data lend further support to the notion that initial surface interactions are governed by general physical principles and suggest that near surface swimming might be a general strategy for target selection employed by different flagellated bacteria.

### During NSS,*S*. Typhimurium stops preferentially at topological obstacles

Analysis of our movies so far suggested that during NSS, rounded cells (e.g. dividing cells) represent preferential sites for stopping. We hypothesized that bacterial stopping can be explained by the prominent topological features of these rounded cells. If so, bacteria should also stop at artificial topological obstacles and bacteria should accumulate at such sites. This was tested in two different ways, i.e. in a simplified experimental setup and by a computer simulation (see below).

**Figure 4:**
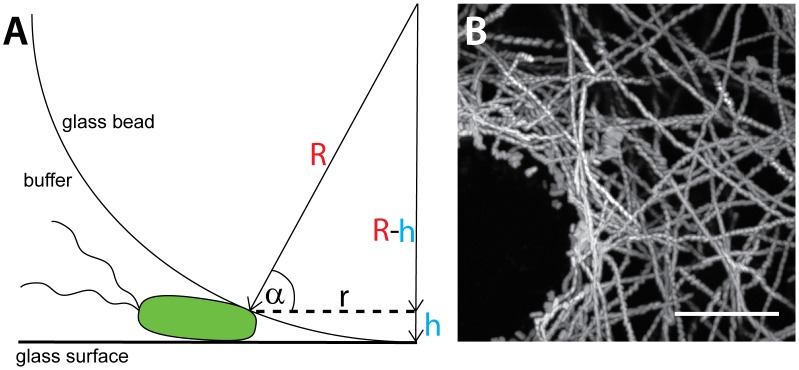
Swimming behavior of *S.* Typhimurium on gelatine coated glass beads. Gelatine coated glass beads (500 µm diameter) were placed into a glass-bottom dish and the swimming behavior of *mCherry* expressing bacteria was recorded by time lapse fluorescence microscopy as described in Fig. 3. (A) Illustration of a bacterium stopping at the glass bottom of a coated glass bead (illustration not to scale). (B) Maximum intensity plot (ImageJ) superimposing all frames of a 15 sec movie acquired at 20 frames per second (300 frames total; suppl. Video S3) illustrating the movement of *S*. Typhimurium (*S*.Tm^wt^(*mCherry*)) within the vicinity of the bead. Scale bar: 10 µm.

In order to experimentally test our hypothesis, we have analyzed the initial surface interactions of bacteria with small glass beads which were placed as artificial obstacles onto a glass surface. Glass-bottom tissue-culture dishes harboring glass beads (Ø = 500 µm) were therefore inoculated with *S*.Tm^wt^(*mCherry*) and bacterial movement patterns were analyzed by time-lapse fluorescence microscopy, as described in Figs. 1, 2 and 3. Again, the bacteria were moving for long distances along the glass surface, but were stopping in the immediate vicinity of the glass beads (Fig. 4A, B; suppl. Video S3). 45–70% of these bacteria continued to swim or took off again before the end of the movie, indicating that these were truly stopping (not binding/docking). Thus, topological obstacles can facilitate site-specific stopping of bacteria engaged in NSS.

In addition, these data allowed a rough estimation of the “altitude” at which *S*. Typhimurium swims above the glass surface. Based on the geometry of the glass bead, the center of the bacterial cell was 0.43+/−0.07 µm away from the surface. Similar results were obtained for beads with smaller diameters (150 and 30 µm, respectively). The rod-shaped *S*. Typhimurium cell has a radius of approx. 0.5 µm. Therefore, most of the “distance” is attributable to the bacterial cell and the NSS “altitude” is most likely <150 nm above the glass surface. These observations were in line with earlier work [27], [28], [29], [34] describing bacterial NSS.

### Modeling of NSS-driven target site selection by *S*. Typhimurium

The data presented above indicated that NSS affects target cell selection in two ways: by increasing the local pathogen density on surfaces and by enhancing the probability of surface contacts (stopping) at topological obstacles projecting from this surface. A computer simulation was used to verify whether these two phenomena are sufficient for explaining target site selection.

We modeled the interaction of *S.* Typhimurium with a three dimensional landscape consisting of a flat surface and one spherical obstacle, partially submerged into the surface (Materials and Methods). The particles (motile, but non-chemotactic “bacteria”) were introduced and moved linearly within the 3D virtual space above the surface. Upon contact with the sphere, the particles were either “stopping” (10% likelihood) or reflected (90% likelihood). Three different scenarios were analyzed with respect to the particles encountering the flat surface. (1) the “random” scenario: particles were reflected back into 3D space and randomly assigned a new direction of movement. (2) the “billiard” scenario: particles were reflected with an angle of reflection identical to the angle of impact. (3) the “NSS” scenario: particles encountering the surface did not leave but followed the surface via NSS. If the sphere was encountered during NSS, the particles stopped at this site with a likelihood of 10%.

**Figure 5:**
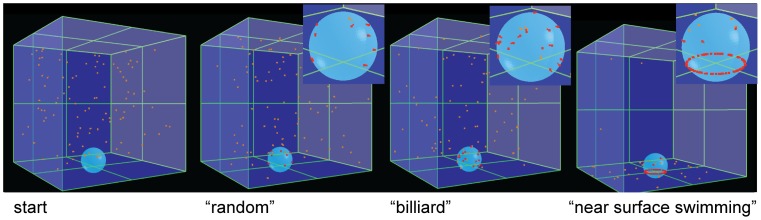
Simulation of *S*. Typhimurium targeting to physical obstacles. (A) To simulate *S*. Typhimurium targeting to physical obstacles, a round “cell” (light blue sphere) and linearly moving “bacteria” (orange particles) were modeled in an *in silico* experiment. If during the simulation a bacterium hits the sphere, it is either deflected or has a 10% chance of remaining at that site (“stopping”, red particles). Behavior of particles on the base of the cage ( = “surface”) and upon hitting the sphere if no docking occurred differed according to the respective scenario: Reflection with a random angle in the “random” scenario, with an angle of reflection identical to the angle of the impact in the “billiard” scenario and no reflection but movements following the surface of the object in the “NSS” scenario. The start of the simulation is depicted in the far left. Inserts show an enlarged view of the area around the sphere.

In the NSS scenario (scenario 3) but not the random or the billiard scenario, particle accumulation occurred right at the topological obstacle (Fig. 5). Therefore, NSS and stopping at physical obstacles are sufficient for explaining the target site selection observed in simplified model systems (Fig. 4A, B) and tissue culture infection experiments (Fig. 1, [31], [32]).

### Docking – as observed in standard infection assays – displays the same target site preference as NSS-mediated stopping

So far, our analyses of the target site preference of *S*. Typhimurium had focused on the initial surface interactions, i.e. landing, take-off, NSS and stopping. Next, it was important to establish how these initial interactions may affect the subsequent steps of the infection process, i.e. docking. If stopping increases the probability of binding and irreversible docking at the respective site, stopping (as observed by time lapse microscopy; Fig. 1, 2) and docking should display an equivalent target site preference.

**Figure 6:**
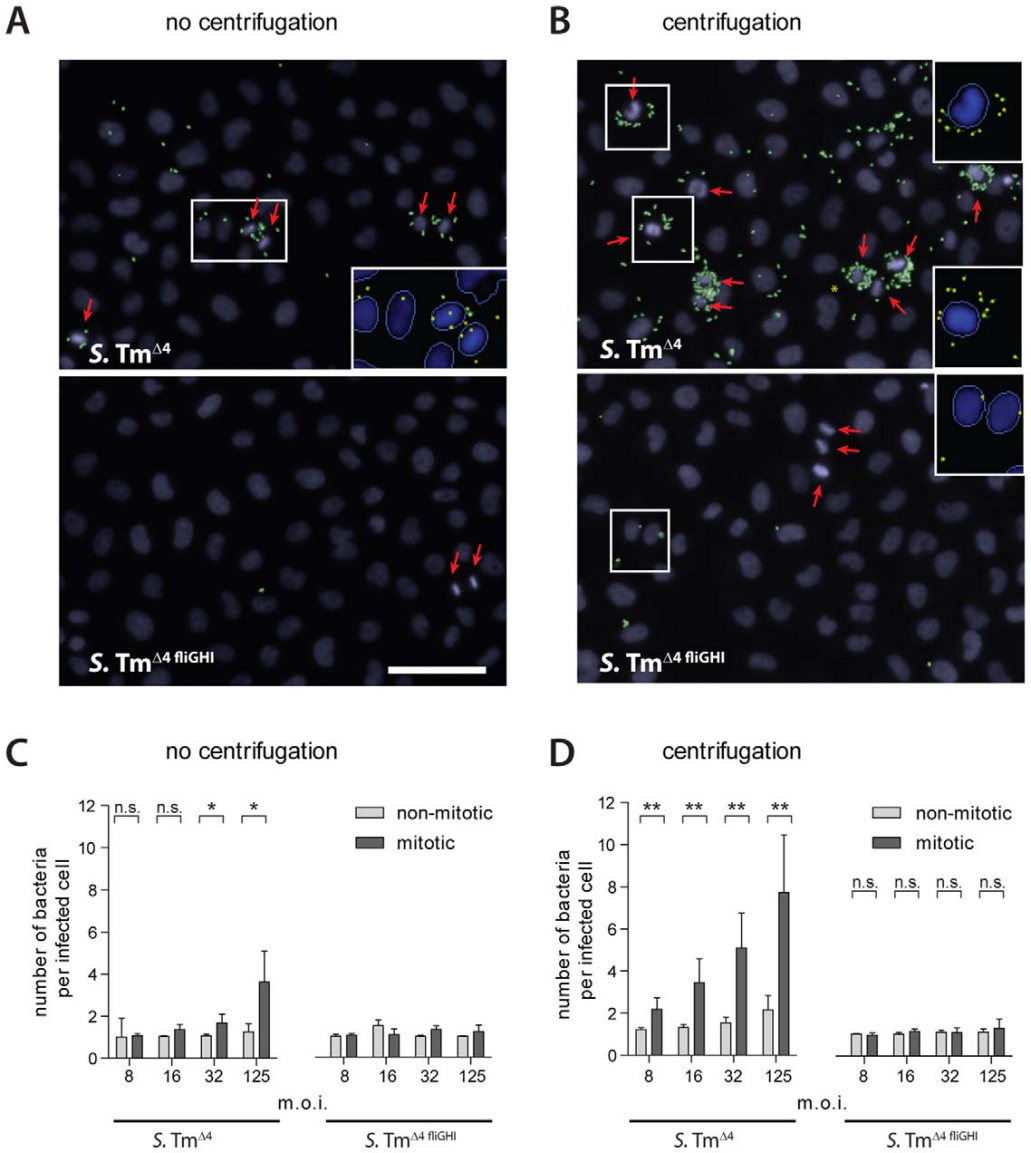
Motility determines the targeting preference of *S*. Typhimurium for mitotic cells. HeLa cells were infected with *S*.Tm^wt^ or *S*.Tm^ΔfliGH^ at the indicated m.o.i. for 6 min either without centrifugation (A, C) or including a centrifugation step for 10 min at 500 g (B, D), followed by staining of nuclei, extracellular bacteria (anti-LPS antibody) and automated microscopy. Using an automated analysis algorithm, nuclei and individual bacteria were detected and mitotic nuclei identified. (A, B). Representative images are shown for infections at an m.o.i. of 125. Nuclei are shown in blue, extracellular bacteria in green. The red arrows indicate mitotic nuclei. For selected parts of the image (white boxes)the automated detection of nuclei and bacteria is shown (see inserts). Please note, that our image analysis algorithm is not able to distinguish all bacteria in highly crowded regions (*S*.Tm^Δ4^ and centrifugation, not shown); therefore, docking onto mitotic cells might be underestimated and our quantitative analysis below would be a conservative estimation. Scale bar: 40 µm. (C, D) Infection efficiency at the respective m.o.i. with respect to the number of docked bacteria per infected cell (non-mitotic cells, light grey; mitotic cells, dark grey). Each bar represents the median. The error bars represent the standard deviation of 6 replicas from 3 independent experiments. Approximately 5000 cells per replica were analyzed. *p<0.05, **p<0.01 (Mann-Whitney-U test). For the immotile strain(*S*.Tm^Δ4 fliGHI^; no centrifugation), we did not detect enough bacteria on mitotic cells for a reliable statistical analysis.

To assess the target site preference of binding/docking on host cell layers quantitatively, we have employed a well-established “standard” infection protocol [9], [32]. HeLa cells were infected for 6 min with *S*.Tm^Δ4^ at the indicated m.o.i., washed gently, fixed and stained (see Materials and Methods). As binding/docking occurred approximately 50-fold less frequently than stopping (see above), we employed higher multiplicities of infection than in the time lapse microscopy experiments. Visual inspection indicated that *S*. Typhimurium has a pronounced targeting preference for rounded cells (Fig. 6A, top panel; “mitotic” nuclei with condensed DNA highlighted in yellow). In particular, the bacteria were found to dock to the base of rounded cells (suppl. Fig. S3A). In order to quantify this phenotype, we employed automated fluorescence microscopy, and an automated image-analysis routine (Materials and Methods).

Rounded cells were targeted (docked) with significantly higher efficiency than non-dividing cells (Fig. 6C, left panel).This targeting preference for rounded cells was also observed by manual quantification (*S*.Tm^Δ4^; suppl. Fig. S3B) and in infection experiments with *S*.Tm^wt^ (data not shown). These findings were quite similar to our observations during the initial surface interactions (Figs. 1 and 2).Rounded mitotic cells seem to represent topological obstacles within the cellular landscape. In fact, the rounded mitotic cells were significantly “higher” than the interphase cells (12±2 µm vs. 5.4±1.1 µm; n = 20 each; p<0.0001, Mann-Whitney-U test). Taken together, NSS-driven stopping and binding/docking displayed equivalent preferences for topological obstacles like rounded cells.

Next, we have addressed the role of flagellar-driven motility. It is well established that flagella are required for cellular invasion of *S.* Typhimurium [15], [16], [17], [18], [19]. The data presented above suggested that flagella-driven NSS might determine the target site preference. In order to study the role of bacterial motility in the targeting preference of binding/docking, we analyzed the host-cell interaction patterns of the non-motile mutant *S*.Tm^Δ4 fliGHI^ (SL1344 *fliGHI*) which does not express flagella. This mutant and other non-motile S. Typhimurium mutants did not dock efficiently at all (Fig. S4). Importantly, the few HeLa cells that were infected harbored just one bacterium, even at high m.o.i. (Fig. 6A, bottom panel; Fig. 6C, right panel). In line with our hypothesis, *S*.Tm^Δ4 fliGHI^ did not display a targeting preference for rounded cells (Fig. 6A,C).

In order to increase the chances of a host cell encounter by *S*.Tm^Δ4 fliGHI^, equivalent infection experiments were performed applying mild centrifugal force (500 g for 10 min) to increase the collision rate between non-motile *S*.Tm^Δ4 fliGHI^ and the host cells (Materials and Methods). This strategy is commonly used for “rescuing” invasion defects of non-motile *S.* Typhimurium mutants, (e.g. [16], [35]). As expected, centrifugation increased the number of binding/docking*S*.Tm^Δ4 fliGHI^ in our assay (Fig. 6B, data not shown). Nevertheless, *S*.Tm^Δ4 fliGHI^ did not display any target preference for the rounded (mitotic) cells, even at the highest m.o.i., tested (Fig. 6D; right panel). This was in line with our hypothesis that flagella-driven NSS determines not only the targeting preference of “stopping”, but also that of binding/docking.

Finally, we analyzed the role of chemotaxis, i.e. directed motility along chemotactic gradients. *S*.Tm^Δ4 cheY^ is an isogenic and motile mutant incapable of chemotaxis (compare Fig. 2E, F). This mutant yielded equivalent results as *S*.Tm^Δ4^including a highbinding/docking efficiency and a pronounced targeting preference for rounded cells (data not shown). Taken together, these results suggested that preferential infection of rounded cells is attributable to simple non-directed motility and that chemotaxis was not required, at least in this simple tissue culture model.

### Altering the host cellular morphology affects the initial surface interactions and docking

During NSS, stopping and docking occurred preferentially at rounded cells. If targeting was indeed dictated by topological obstacles, manipulation of the host cell morphology should affect both, the initial surface interactions and docking by *S*. Typhimurium. To test this hypothesis, we manipulated the host cellular morphology with cytochalasin D (Cyt. D). This drug depolymerizes actin filaments causing the cells to round up.

**Figure 7:**
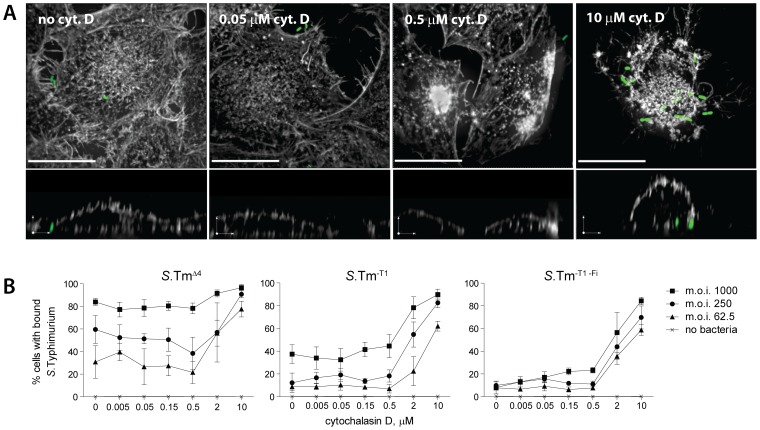
Cellular morphology affects docking of *S*. Typhimurium. (A) Cells were incubated with the indicated concentration of cytochalasin D for one hour prior to infection with*S*.Tm^Δ4^(pGFP) at an m.o.i. of 125 for 10 min. Afterwards, cells were fixed as described in Fig. 6 and stained with DAPI and TRITC-phalloidin (stains actin). Stacks of confocal images were acquired in the actin channel (grey) and the bacteria channel (green). An extended focus projection and a reconstruction of a zx-layer are shown. Scale bar, upper images: 20 µm, zx-layer: 5 µm. (B)Cells were pre-treated with the indicated concentration of cytochalasin D for 1 hour and infected with the respective *S.* Typhimurium strain for 12 min. After washing, fixing and staining *S.* Typhimurium docking was quantified by an automated microscopy based docking assay [9]. The curves shown summarize 3 independent experiments. Error bars: standard deviation.

First we analyzed the effect of cytochalasin D treatment on binding/docking. HeLa cells, pretreated with the indicated concentration of cytochalasin D for one hour, were infected for 12 minutes with *S*.Tm^Δ4^(pGFP), *S*.Tm^-T1^(pGFP) or *S*.Tm^-T1-Fi^(pGFP) at the indicated m.o.i..Afterwards, cells were fixed and stained and we analyzed the actin-based cytoskeleton and bacterial docking (Materials and Methods). Since the automated evaluation now focused on the percentage of infected cells (not the number of individual bacteria per infected cell), overlapping but somewhat higher m.o.i.s were used than in Fig. 6. As shown in Fig. 7A, HeLa cells were partially rounded at 2 µM and fully rounded at 10 µM cytochalasin D. All three *S.* Tm strains bound/docked to the rounded cells with an increased efficiency (Fig. 7B). Equivalent results were obtained with another actin-disrupting drug (latrunculin B; suppl. Fig. S5). This verified that the host cell topology has a profound effect on the target site preference of binding/docking by *S*. Typhimurium.

**Figure 8:**
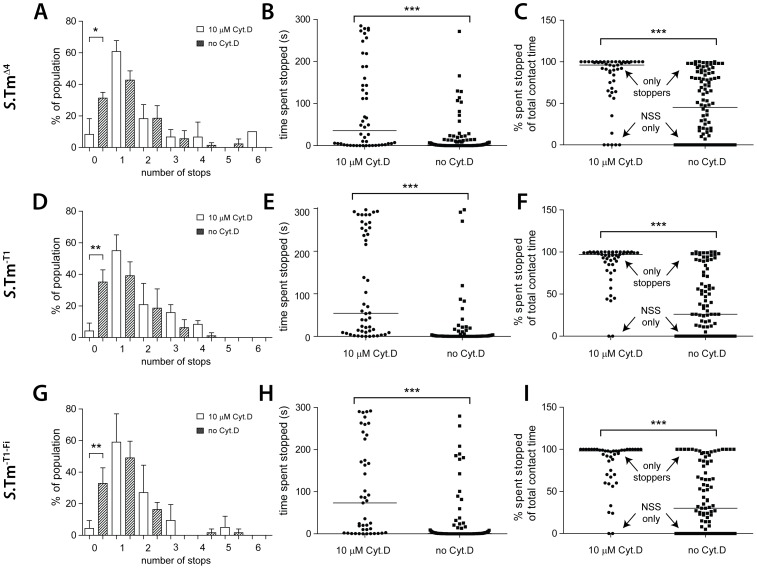
Host cell morphology affects the NSS pattern of *S*. Typhimurium. HeLa cells were either treated with 10 µM cytochalasin D or mock-treated and infected with *S*.Tm^Δ4^(pGFP), *S*.Tm^-T1^(pGFP) or *S*.Tm^-T1-Fi^(pGFP), as indicated at an m.o.i. of 1.5. The infection process was imaged for 5 min by time-lapse fluorescence microscopy. Data for bacteria without cytochalasin D were taken from Fig. 1D–F and Fig. 2A–D and are displayed for comparison. (A), (D), (G) Fraction of bacteria making no stops or the indicated number of stops on the cell layer (compare Fig. 1E). The effect of cytochalasin D on the number of bacteria with no stops vs. one or more stops was statistically significant for all 3 strains (Fisher's exact test). (B), (E), (H) Cumulative time a single bacterium spent stopped over the entire duration of the contact with the cell layer. (C), (F), (I) Fraction of the contact time that a given bacterium spent stopped; the remaining percentage of time was spent in NSS. *: p<0.05; **: p<0.01; ***: p<0.0001.

Next, we analyzed the effects of the cytochalasin D treatment on the initial surface interactions. HeLa cells pretreated with 10 µm cytochalasin D were infected with *S*.Tm^Δ4^(pGFP), *S*.Tm^-T1^(pGFP) or *S*.Tm^-T1-Fi^(pGFP) and landing, NSS, stopping and take-off were analyzed by time-lapse microscopy as described in Fig. 1 and Fig. 2. Strikingly, on the cytochalasin D-treated cells, nearly all bacteria engaging in NSS made at least one stop. Only very few bacteria displayed uninterrupted NSS (“NSS only”; Fig. 8A, D, G). This was quite different from our data on untreated HeLa cells where 33% of all bacteria displayed uninterrupted NSS (see Fig. 1E). Furthermore, on cytochalasin D-treated cells, the time spent at each stop was significantly longer and some of these bacteria “stopped” for the entire course of the 5-min experiment (Fig. 8B,E,H). In a similar analysis, the fraction of time that*S*.Tm^Δ4^(pGFP), *S*.Tm^-T1^(pGFP) or *S*.Tm^-T1-Fi^(pGFP) spent stopping at the host-cell surface was significantly higher than in untreated HeLa cells. In fact, on the cytochalasin D-treated cells, a majority of the bacteria spent most of their time “stopping” (“only stoppers”; Fig. 8C,F,I). Again, no differences were observed between *S*.Tm^Δ4^(pGFP), *S*.Tm^-T1^(pGFP) and *S*.Tm^-T1-Fi^(pGFP), confirming that “classical” adhesins do not significantly affect these transient initial bacteria-host cell interactions. In conclusion, the shape of host cells has a profound effect on initial surface interactions and binding/docking. This provides further evidence that NSS leads to a target site preference for physical obstacles.

### Membrane ruffles represent physical obstacles mediating stopping, binding and docking

Membrane ruffles triggered by the TTSS-1 virulence system represent a well-known topological obstacle encountered on infected cell layers [36], [37], [38], [39]. We hypothesized that membrane ruffles might enhance local stopping, binding and docking at pre-existing ruffles, thus leading to cooperative invasion.

**Figure 9:**
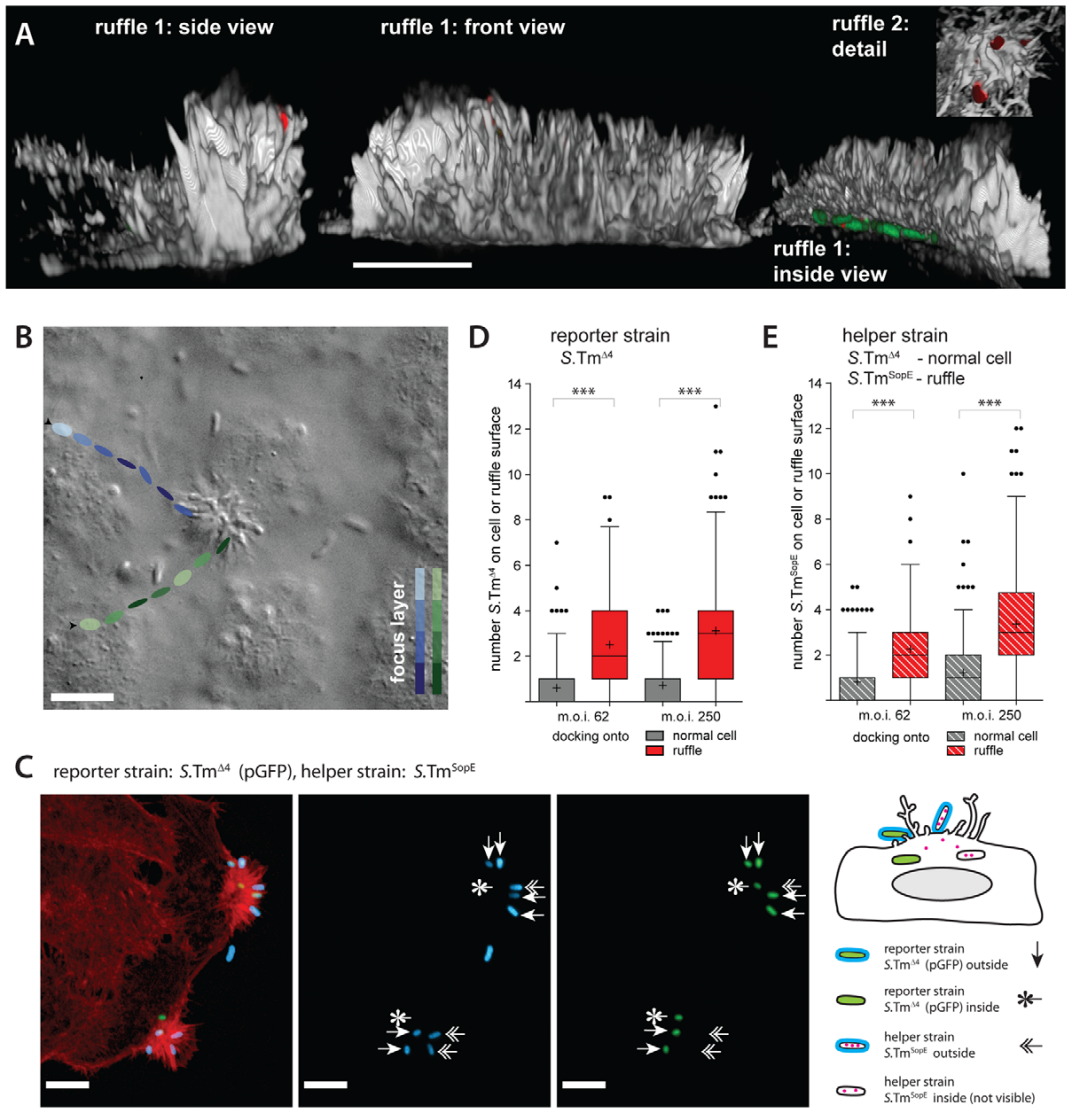
Ruffles are a site of cooperative invasion. (A) 3D-reconstruction of ruffles on HeLa cells infected for 6 min with *S*.Tm^SopE^(pGFP) at an m.o.i. of 250. The actin channel (phalloidin stain) is shown in grey, *S.* Typhimurium in green (GFP) and extracellular bacteria in red (anti-*Salmonella* LPS stain before permeabilization). 3 views of a typical, large ruffle (ruffle 1) and details of another ruffle (ruffle 2) are depicted. Scale bar: 10 µm. (B) HeLa cells were infected with a 1∶1 mixture of *S*.Tm^SopE^ and *S*.Tm^Δ4^(pGFP)at an m.o.i. of 5 and a movie was acquired via DIC imaging (see also supplementary VideoS4). A snapshot of the movie is depicted. The tracks of 2representative bacteria and their estimated position in the z-axis are indicated by the colored ellipsoids. Both bacteria stop at the ruffle and stay for the remaining observation time (blue track: 4.95 s, green track: 1.24 s). Scale bar: 9 µm. (C) Quantification strategy for analyzing *S*. Typhimurium docking onto ruffles. HeLa cells were infected for 6 min with a 1∶1 mixture *S*.Tm^Δ4^(pGFP) (green; reporter strain), shown in green and the *S*.Tm^SopE^ as a helper strain which did not express *gfp* at an m.o.i. of 62.5 for each strain. After infection, cells were fixed and stained for actin (TRITC-phalloidin, red) and extracellular *S.* Typhimurium (indirect immunofluorescence using an anti-*Salmonella* antibody; blue; stained before permeabilization). Three types of *S.* Typhimurium can be distinguished: Extracellular reporter *S*.Tm^Δ4^ (labeled green and blue), extracellular helper *S*.Tm^SopE^ (only blue), and intracellular reporter *S*.Tm^Δ4^ (only green). Intracellular helper *S*.Tm^SopE^ is non-fluorescent and cannot be detected. Scale bar: 10 µm.(D, E) HeLa cells were infected for 6 min with a 1∶1 mixture of a helper strain (either *S*.Tm^Δ4^ or *S*.Tm^SopE^) and the reporter strain *S*.Tm^Δ4^(pGFP) at the indicated m.o.i.. Cells were stained for actin and extracellular bacteria were stained with anti-LPS antibodies. In the control scenario (helper strain *S*.Tm^Δ4^, only non-ruffling cells) bound bacteria were quantified for the area of a whole cell (grey bars); in the ruffling scenario (helper strain *S*.Tm^SopE^) bacteria were quantified over the area of a ruffle as explained in panel (B,C).Even with a complex 3D structure, the surface of a ruffle should be much smaller than the surface of a whole cell. Therefore, if anything, our approach should underestimate the specific recruitment of bacteria onto ruffles. Extracellular bacteria of the reporter strain and the helper strain were quantified separately ((D): reporter strain, expresses *gfp*; (E): helper strain; no *gfp*). The bars summarize 170–220 cells/ruffles from two independent experiments. ***: p<0.0001.

To study the targeting of ruffles, we have focused on binding and docking. These two steps of the infection process can be analyzed using “standard” fluorescence microscopy assays [9], [32]. First, we employed a co-infection strategy using a “helper strain” and a “reporter strain”. *S*.Tm^SopE^ (without plasmid) was chosen as the helper strain, since its effector SopE is able to trigger pronounced membrane ruffling [5], [32]. *S*.Tm^SopE^ carries deletions of the effectors *sipA*, *sopB* and *sopE2*, thus eliminating confounding pleiotropic actions of these effectors on the host cell. SopE-induced ruffles appeared within 5 minutes, are known to have a prominent shape, and represent a large physical obstacle on an otherwise much flatter cellular surface (Fig. 9A; [40]). *S*.Tm^Δ4^ (pGFP), which does not trigger ruffles itself, was used as a “reporter” to assess docking to the ruffles. In a time lapse microscopy experiment employing DIC and fluorescence imaging both strains engaged in NSS and stopped frequently at ruffles (Fig. 9B; supplementary VideoS4; data not shown).

To analyze a preference of *S*. Typhimurium for cellular ruffles in a quantitative manner, HeLa cells were infected with a 1∶1 mixture of *S*.Tm^SopE^ and*S*.Tm^Δ4^(pGFP) at a high or a lower m.o.i for 6 min. In control experiments (no ruffles), HeLa cells were infected with a mixture of *S*.Tm^Δ4^ and *S*.Tm^Δ4^(pGFP). Subsequently, cells were fixed followed by staining of extracellular bacteria (using an anti-*Salmonella* antibody; Materials and Methods). After permeabilization of the cell membrane, the actin cytoskeleton was stained. The data evaluation strategy is depicted in Fig. 9C.In the experiments using *S*.Tm^SopE^ as the helper strain (ruffling occurs), we determined the number of*S*.Tm^Δ4^ (pGFP) (Fig. 9D, red bars) and *S*.Tm^SopE^(Fig. 9E, red bars) residing on the respective ruffle. In the negative controls (no ruffling; co-infection with *S*.Tm^Δ4^ and *S*.Tm^Δ4^ (pGFP)) we quantified all bacteria located on the respective cell(Fig. 9D, D grey bars). Comparing targeting to a ruffle as opposed to targeting to a whole cell (control w/o ruffle) is a conservative strategy for detecting ruffle-specific target site preferences, since the whole cell has a much larger area than an individual ruffle. Furthermore, it should be noted that the anti-*Salmonella* antibody was applied before permeabilization (Materials and Methods). This allowed us to discern internalized and external reporter bacteria.

In all experiments, *S*.Tm^SopE^ and *S*.Tm^Δ4^(pGFP) docked more efficiently to ruffles than to non-ruffling cells (Fig. 9D, E compare red and grey bars). This was true for the reporter strain (Fig. 9D; *S*.Tm^Δ4^ (pGFP)) as well as for the helper strain (Fig. 9E; i.e. *S*.Tm^SopE^). A similar effect was also observed in 12-min infection experiments (data not shown). Therefore, ruffling stimulated docking of bacteria to cellular ruffles. Moreover, ruffling facilitated internalization of *S*.Tm^Δ4^(pGFP).Internalized *S*.Tm^Δ4^ (pGFP) was detected in all co-infections with *S*.Tm^SopE^ (1.4 and 1.6 bacteria per ruffle on average for the experiment in Fig. 9D for m.o.i.s of 62 and 250, respectively). No internalized*S*.Tm^Δ4^ (pGFP) was detected in the negative controls (no ruffles; *S*.Tm^Δ4^ as helper strain). Equivalent data were obtained in an analogous experiment using automated image acquisition and analysis methods (suppl. Fig. S6). In another control we tested immotile bacteria (*S*.Tm^Δ4 fliGHI^ (pGFP)). As expected, these bacteria did not attach to ruffles or normal cells (Fig. S7). Again, cellular attachment was rescued upon centrifugation; however, this did not lead to any targeting preference for ruffles vs. non-ruffling cells (Fig. S7). Finally, we explored the targeting to ruffles by *E.coli* Nissle, a non-invasive motile bacterium. In co-infection experiments with *S*.Tm^wt^, *E.coli* Nissle displayed a targeting preference for host cellular ruffles (Fig. S8).

Taken together, the effector protein SopE is required for triggering ruffles. Once the ruffles are formed, they seem to represent prominent physical obstacles which facilitate stopping, binding, docking and internalization of motile pathogens. However, please note that we cannot exclude that other factors, besides ruffle topology (e.g. altered membrane structure or composition in the ruffle) might also contribute to *S*. Typhimurium targeting to these sites.

### Cooperative invasion at ruffles

**Figure 10:**
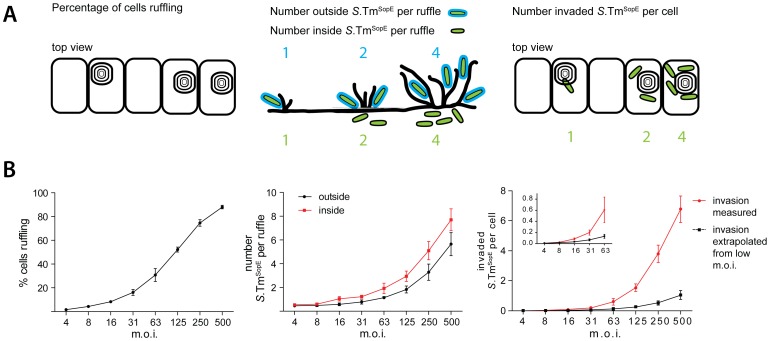
Cooperative invasion at membrane ruffles. (A) Scheme explaining our quantification strategy for cooperative invasion. (B) HeLa cells were incubated with *S*.Tm^SopE^(pGFP) at the indicated m.o.i. for 9 min, followed by washing, fixation and staining of actin and extracellular bacteria (anti-LPS antibody; staining before permeabilization). Left panel: quantification of the fraction of cells carrying ruffles. Middle panel: quantification of the number of “inside” and “outside” *S*.Tm^SopE^(pGFP) per individual ruffle. Right Panel: number of invaded *S*.Tm^SopE^(pGFP) per cell, calculated by multiplying the number of intracellular bacteria per ruffle with the fraction of ruffling cells. The black line indicates the estimated number of invaded *S*.Tm^SopE^(pGFP) assuming independent invasion events, extrapolated from experimental data of ruffles with only one associated bacterium at the lowest m.o.i.. Each data point summarizes 4×150 cells for the analysis of cellular ruffling and 4×25 cells for inside and outside bacteria from 2 independent experiments. Error bars: standard deviation.

Finally, we reasoned that *S.* Typhimurium recruitment onto ruffles might lead to cooperative invasion. If multiple bacteria dock to the same ruffle, more effector proteins are delivered, thus increasing the ruffle size and enhancing the chance for stopping, binding and docking of additional bacteria. To test this hypothesis, HeLa cells were infected for 9 min with *S*.Tm^SopE^(pGFP) at increasing multiplicities of infection, fixed and stained. We first quantified the fraction of ruffling cells (Fig. 10A, B, left panel).Next we determined(at each m.o.i.)the number of intracellular and extracellular bacteria residing in an individual ruffle (Fig. 10A, B, middle panel). Finally, we determined the number of “invaded *S.* Tm^SopE^” per cell (Fig. 10 A, B, right panel). This was achieved by multiplying the percentage of ruffling cells with the number of intracellular bacteria per ruffle. All these parameters increased as a function of the m.o.i.

Next we wanted to determine the invasion efficiency mediated by a single bacterium without “support” from other bacteria. We therefore focused at low m.o.is., where typically one bacterium was observed per ruffle. The few ruffles with two associated bacteria were excluded from the subsequent analysis. The single bacterium was either located outside or within the host cell (Fig. 10B, m.o.i. 4 and 8). To estimate invasion efficiency by this single bacterium alone without possible support by other bacteria, this invasion efficiency was extrapolated to higher m.o.i., assuming a linear increase of invasion efficiency with increasing m.o.i. (Fig. 10B, right panel, black line). Strikingly, this extrapolated invasion efficiency was much lower than the observed invasion efficiency at higher m.o.i. (Fig. 10B, right panel, red line).This indicated that *S.* Typhimurium invasion occurred in a cooperative fashion, most likely by provoking stopping, binding and docking of additional bacteria engaged in NSS at sites of membrane ruffling.

## Discussion

The mechanism of target site selection by *S*. Typhimurium had remained enigmatic. We have analyzed the initial surface interactions between the pathogen and the host by time-lapse microscopy, by comparative analysis of pathogen movements on cells and glass surfaces as well as by standard infection experiments. Upon encounter with a host-cell layer, we identified a distinct early phase of the infection characterized by landing, near surface swimming and stopping, which preceded later events such as *fim*- or TTSS-1-mediated binding and docking. In this initial phase, flagellar motility has at least two functions in establishing the contact with the host cell, i.e. propelling the pathogen towards the host cell layer and facilitating NSS. NSS is likely attributable to physical forces emanating from the pathogen movement along the surface. Here we found that NSS increased the local pathogen concentration on the host-cell surface, and lead to stopping at topological obstacles. NSS thereby mediates preferential docking at the base of rounded cells and on pre-existing membrane ruffles. Thus, the target-cell selection for dividing cells and cooperative infection at membrane ruffles can be explained simply by physical forces between the host cellular surface and the motile pathogen, which govern the initial phase of the pathogen-host cell interaction.

Different models have been proposed to explain NSS [27], [28], [41], [42], [43], [44]. Recent approaches have combined hydrodynamic entrapment and DLVO interactions with Brownian motion, which leads to random changes of the bacterial NSS-altitude [37]. Random variations in surface distance also lead to predictable changes of the angle of bacteria towards the surface and the radius of the curved track. Therefore, Brownian motion can enable probing of the surface following different curvatures at different heights when the bacteria are swimming along. This variability may help to further expand the surface probing capacity of a motile pathogen [37]. However, it should be noted that the Brownian motion-forces are much weaker than the thrust provided by the flagella. This may explain why stopping and docking on open glass surface areas is much less frequent than at the base of elevated obstacles located on the surface. Only at these obstacles, the force provided by the flagella is fully employed to counteract repulsive forces thus driving the bacterium as close to the surface as possible. This may increase the chances for the formation of stable contacts as required for binding and docking.

Are “stopping” and “docking” related? In both cases, *S*. Typhimurium stays at one particular spot on the host cell surface for at least some time. However, the retention mechanisms seem to differ. Docking and binding, i.e. long-term association with host cells are mediated by adhesins [45], [46]. In the case of HeLa cells, docking is mediated mainly viaTTSS-1 and type-1 fimbriae which is why the mutant strain*S*.Tm^-T1-Fi^ has a reduced docking efficiency [9]. In contrast, stopping was not significantly affected in the case of*S*.Tm^-T1-Fi^, implying that neither TTSS-1 nor type-1 fimbriae mediate stopping (Fig. 2). Furthermore, washing (as performed in docking experiments; see Materials and Methods), removed ≥98% of all “stopped” bacteria from the host cell surface. Presumably, the remaining ∼2% were docked, suggesting that stopping is mediated by a weaker force and that it is approximately 50-fold more frequent than docking, at least during the first minutes of infection. In spite of these differences, both stopping and docking required motility (but not chemotaxis) and occurred with high probability at rounded cells and membrane ruffles. Based on these considerations, we propose that stopping at topological obstacles may simply extend the residence time at a given location (close to the surface), thereby increasing the probability of adhesin-mediated binding and docking. It is tempting to speculate that Brownian motion might randomly drive stopping bacteria into even closer proximity of the host cell surface, thus increasing the chances for a successful engagement of TTSS-1 or type-1 fimbriae [34]. In this way, prolonged stopping and Brownian motion would foster the preferential infection of rounded cells and cell ruffles. Thus, landing, NSS and stopping may allow prolonged probing ata very limited area of the 2D surface. If docking is unsuccessful, *S*. Typhimurium continues NSS or takes off into and may engage in initial surface interactions at another site.

NSS recruits *S*. Typhimurium into membrane ruffles, thus promoting cooperative invasion. Invasion of more than one bacterium at a single ruffle has been described before [47], [48], [49] but no mechanistic explanation and no evidence of cooperativity has been provided. Our data show that ruffles represent topological obstacles favoring stopping and productive invasion. Therefore, ruffles might be regarded as a site of “communication” between individual bacteria. Firstly, this implicates the exploitation of invasive strains by non-invasive strains, which can subsequently invade. This type of “rescue” has been described previously [37], [50]. Secondly, it implicates cooperation between invasive strains, in two respects. When *S*. Typhimurium makes a favorable docking interaction and induces membrane ruffling in the host cell, this increases the chance that additional bacteria can “find” this invasion-permissive site. Furthermore, in some hosts or cell types, higher dosages of effector proteins might be required for triggering successful invasion. In this case, recruitment into ruffles might allow larger amplitudes of stimulation. Certainly, this would be of importance for the mechanistic interpretation of results from tissue-culture cell-infection experiments.

In animal experiments, flagella and motility are also required for efficient gut infection [21], [22], [24], [51].Here, the flagella serve additional functions not observed in tissue culture. In the inflamed gut, flagella facilitate chemotactic movement thus mediating access to the nutrient-rich molecules secreted by the gut wall [24]). Thereby, chemotactic motility propels the pathogen towards the gut surface. For this reason, both, chemotaxis and motility are required in the gut [24]. This additional function of flagella, which does not affect the tissue-culture infection, has precluded straightforward animal-infection experiments addressing the “within host” importance of the NSS-based, chemotaxis-independent targeting mechanism described here.

Does NSSor stopping also occur at the gut surface? In this study we demonstrated NSS *in vitro* at the surface of HeLa cells and polarized epithelial cells. However, in previous studies, *S*. Typhimurium was found to accumulate at the surface of the gut epithelium during the first phase of the infection [24]. Furthermore, *S*. Typhimurium swimming along the epithelial surface has also been visualized by in vivo live microscopy in the cecum of infected mice [6]. At 4 h post infection, the pathogen was found to swim along the surface of infected crypts at a speed of 5–50 µm/sec. Subsequently bacteria stopped at the epithelium and entered into enterocytes. While these experiments did not elaborate on the individual phases of the initial surface interactions, they clearly demonstrate that NSS occurs within the host's intestine. Later on, upon triggering mucosal inflammation, *S*. Typhimurium seems to invade into neutrophils which are transmigrating in large numbers into the gut lumen [52]. Intriguingly, neutrophil infection was found to require motility. It is tempting to speculate that neutrophils are infected by *S*. Typhimurium during transmigration. While crossing the epithelial barrier, the luminal part of the neutrophil may form a physical obstacle stopping *S*. Typhimurium swimming along the epithelial surface. These observations suggest that NSS-mediated targeting of host cells may occur in vivo. A detailed analysis of these processes during the course of a real infection will be an interesting topic for future research.

The NSS-mediated targeting mechanism identified in our work is based on general physical forces which act on any particle moving on a 2D surface. Therefore, it should pertain to many other motile bacteria, including pathogens (e.g. enteropathogenic *E. coli*, *Yersinia* spp.), commensals, as well as environmental bacteria. Accordingly, we found that the motile strain *E. coli* Nissle can engage in NSS and target *Salmonella*-induced membrane ruffles. Our results imply that the NSS-mediated targeting should result in preferential binding to physical obstacles present on the respective surface, including abiotic obstacles as well as prominent topological features of surface-exposed cells of a given host. Thus, the initiation of biofilm formation and the infection of animals at particular sites might be governed by the same basic principles. Deciphering these principles will be of great interest for basic microbiology and might allow the development of countermeasures prohibiting the initial steps of bacterial colonization.

## Materials and Methods

### Bacterial strains

Published bacterial strains are listed in Table 1. The construction of additional strains is described, below.

### Time lapse microscopy

Cells were seeded into glass-bottomed culture dishes (Mat Tek) in DMEM (Invitrogen), 10% FCS (Omnilab) containing streptomycin (50 µg/ml), 24 hours prior to the experiment at 300 000 cells (HeLa Kyoto, if not mentioned otherwise) per 35 mm well; experiments were performed in HBSS (Invitrogen), 10% FCS, 20 mM Hepes (Invitrogen), pH 7.2–7.5 (Invitrogen). After exchange of media to HBSS, cells were incubated at 37°C, 5% CO_2_ and infected with the indicated *S.* Typhimurium strain (derivative of SL1344, [53]) carrying plasmid pCJLA-GFP at an estimated m.o.i. of 1.5.

If not stated otherwise, movies were acquired on a Leica DMI-6000B microscope, either using the differential interference contrast mode or the fluorescence mode. DIC-movies were acquired using a 63× oil objective (HCX PLAN Apochromat from Zeiss, NA 1.4); for fluorescence imaging, a 20×-objective (HC PLAN Apochromat from Zeiss, NA 0.75) with a 2-fold optovar was used. If not indicated otherwise, movies were acquired at 10 frames per second for 5 minutes. Movies were analyzed using the program Volocity (Improvision, UK) and the manual tracking mode.

For Fig. 4, we used a Zeiss Axio Observer equipped with a spinning disc confocal head and a 100× objective (oil NA1.4,). For suppl. Fig. S1, we used a Zeiss Axiovert 200 m inverted microscope equipped with an Ultraview confocal head (Perkin Elmer) and a krypton-argon laser (643-RYP-A01 Melles Griot, The Netherlands) and a 20× objective, 0.75NA (Optovar 1.6). In these experiments estimating the influence of gravity, glass-bottom culture dishes containing HBSS but no cells were inoculated with bacteria as described for the other time lapse experiments;

For testing of bacterial near surface swimming on MDCK cells, cells were seeded in glass-bottom culture dishes in DMEM, supplemented with 10% FCS, grown to confluence and let polarize for five days. Infection with *S*.Tm^Δ4^ was done exactly as described for HeLa cells. Movies were acquired on a Leica DMI-6000B microscope using a 20×-objective with a 1.6 optovar. The movie was acquired for 5 min at 10 frames per second and an overlay of the movie is shown.

*S*. Typhimurium strains were grown before infection as described [32]. *E. coli* Nissle was grown identically except that normal Luria-Bertani media was used.

### Quantitative analysis of movie sequences

For quantification of the contact of *S*. Typhimurium with the cells, the interaction was divided into 4 phases: 1) landing: the time of appearance of a *S*. Typhimurium until interruption of the continuous downward movement, indicated by changes in focus, direction and speed. 2) stopping: episodes without changes in any direction for at least 3 frames. 3) take off: the continuous upward movement until disappearance. 4) NSS: a continuous movement in the xy-direction other than 1) or 3). *S*. Typhimurium that left the field of view during quantification, as well as a few cases with ambiguities were excluded.

For quantification of the number of *S*.Tm^Δ4^(pGFP) within the focus depth, either at the cell surface or >100 um above the surface (“in solution”), 40 time points from 2 independent experiments were analyzed. The same time points were used for each movie analyzed and they were spaced throughout the duration of the movie. For each bacterium within focus depth at the specific time point, the entire contact time spent on the surface was analyzed. Each bacterium was thus either counted as engaged in “NSS” or “NSS and stopping”. For the quantification of bacteria swimming in solution, any non-motile bacteria were excluded.

For quantification of the distance, time and speed of *S*.Tm^Δ4^(pGFP) on the glass and cell surface, 20 bacteria from 2 independent experiments were manually tracked from their first contact with the cell to their last contact with the cell (i.e. NSS quantification excluding the landing and take-off stages) or until they left the field of view. *S*.Tm^Δ4^ were selected at random from those arriving in the center of the field of view. The time indicated is NSS-time only and does not include any intermittent time spent stopping at the surface.

### High-resolution microscopy

For high-resolution images, HeLa cells were seeded on glass cover slips for 24 hours and infected with *S*. Typhimurium carrying plasmid pM965 for constitutive *gfp* expression at the indicated m.o.i.. After fixation, *S*. Typhimurium were stained by indirect immunofluorescence using an anti-LPS antibody (Difco) and goat anti-rabbit-Cy5 (Jackson) as a secondary antibody; the actin cytoskeleton was stained by tetra methyl rhodamine isothiocyanate (TRITC)-phalloidin after 5′ permeabilization with 0.1% Triton Tx100. Images were taken on a Zeiss Axiovert 200 m inverted microscope equipped with an Ultraview confocal head (PerkinElmer) and a krypton argon laser (643-RYP-A01, Melles Griot) using a 100× oil immersion objective (PLAN-Apochromat Zeiss with an NA of 1.4). Stacks of 0.2 µm were acquired; deconvolution was performed in the actin channel with the program Volocity and a calculated point spread function. 3D-reconstruction and reconstruction of zx-layers was done using Volocity.

### Automated analysis of *S*. Typhimurium docking

Docking experiments were done as described [9]. In brief: cells were seeded in 96-well micro-clear plates, (half size, Greiner), at 6000 cells per well 24 hours prior to the experiment and infected with the indicated *S*. Typhimurium strain followed by fixation, staining of nuclei and bacteria using DAPI and an anti-*Salmonella* antibody (Difco), respectively. Images were acquired on a MD-Image Xpress microscope (Molecular Devices) using a 4×-objective in the DAPI- and *Salmonella*-channel. Images were analyzed using the open source program CellProfiler [58] and customized Matlab-scripts, available upon request.

### Automated analysis of individual docked bacteria onto mitotic and interphase cells

For automated analysis of individual docked bacteria (Fig. 1) the analysis consisted of three major steps. First, cells and nuclei were detected using the program CellProfiler [58] and cellular properties, such as morphology, texture and intensity were extracted. Second, single bacteria were identified using the “a trous” wavelet transform [59] and every bacterium was assigned to the corresponding host cell. In a third step, mitotic cells were distinguished from interphase cells using supervised machine learning technique. For this purpose, the “Advanced Cell Classifier program” was applied and the classification was performed using the extracted cellular features with an artificial neural network classifier [60]. Finally the average amount of bacteria on mitotic and interphase cells was calculated.

### Manual quantification of *S*. Typhimurium docking and cooperative invasion

Docking onto mitotic cells: HeLa cells were seeded in 96-well Microclear plates (full size, Greiner) at 6000 cells per well 24 hours prior to infection. After infection and fixation, extracellular *S*. Typhimurium was stained using an anti-*Salmonella* LPS antibody and a Cy5-labelledsecondary antibody (Fig. 9). Due to the bright staining of extracellular bacteria, the unique bacterial shape and the high resolution used for quantification bacterial staining was clearly distinguishable from actin staining. For illustrative purposes (Fig. 9C), in one experiment bacteria were stained using a Cy3-labelled secondary antibody. Subsequently, nuclei and actin were stained using DAPI and TRITC-phalloidin, respectively. Finally, bound *S*. Typhimurium bacteria were manually quantified using a 40×-objective (EC Plan-Neofluar objective with a NA of 1.3).Docking onto ruffles: The *gfp*-labeled *S*. Typhimurium strain and the helper strain were mixed at equal dilutions prior to infection. Staining and fixation was done as described for mitotic cells. Testing of cooperativity: *S*.Tm^SopE^-carrying plasmid pM965 was incubated at the indicated concentration with HeLa cells; fixation and staining was done as described. To calculate invasion efficiency of *S*. Typhimurium without cooperativity, invasion was calculated at the lowest m.o.i., excluding ruffles with more than one associated *S*. Typhimurium. At higher m.o.i., this number was assumed to increase proportionally with the number of added *S*. Typhimurium.

### Modeling of *S*. Typhimurium targeting

In our calculations we modeled a three-dimensional environment within a cubic space. A round sphere was placed into this space; the radius of the sphere was chosen to be 1/10 of the length of the surrounding cube. The sphere was partially submerged into the bottom surface of the cube and 75% of the height of the sphere remained within the cube. In addition, 100 single particles were modeled into the cubic space. For simplicity the size of the particles was assumed to be zero in all dimensions. The whole volume of the cube was assumed to be accessible to the particles except the interior of the sphere. All particles were assigned the same constant speed but a randomly chosen vector of movement. The positions of the particles where integrated after travelling a fraction of 1.2*10^−5^ of the length of the square. Upon hitting the bottom surface, particles followed the rules according to the chosen scenario:1) Reflection at a randomly chosen angle in the “random” scenario, where the new vector of movement was randomly chosen (“impossible” movements, for instance into the surface were excluded). 2) Reflection with an angle identical to the angle of infliction in the “billiard” scenario, the vector of “take off” thus mirroring the vector of “landing” or 3) particles started swimming along the surface in the “NSS” scenario. In this scenario the z-vector of movement was set to zero (particles thus following the surface); the x and y-direction of movement remaining unchanged. When encountering the remaining 5 limiting surfaces of the cube, particles were simply reflected. Upon hitting the limits of the sphere, particles had a 10% chance of being attached (simulating stopping/docking); otherwise particles were simply reflected. Particles hitting the sphere during NSS also stopped/docked to the sphere with a likelihood of 10%. Upon each change of direction the movement vectors were adjusted to achieve a constant overall velocity. After an identical number of calculated increments of particle movements the simulation was interrupted and screenshots were acquired.Scatter3D is implemented in C++ and has previously been used to simulate light scattering [61]. Calculations were performed on a desktop computer. Further details are available upon request.

### Construction of strains and plasmids

Strains *S.*Tm^Δ4 fliGHI^, *S.*Tm^-T1 fliGHI^, *S.*Tm^-T1 -Fi fliGHI^ were constructed by P22 transduction [62] of the tetracycline allele of SB245 (*sipABCDsptP::aphT, fliG/H::Tn10*, K. Kaniga and J.E. Galan, unpublished data) into strains *S.*Tm^Δ4^, *S.*Tm^-T1^ and *S.*Tm^-T1 -Fi^, respectively. flgK was deleted in SL1344 (SB300) as described in [35]. Strains *S.*Tm^Δ4 flgK^, *S.*Tm^-T1 flgK^, *S.*Tm^-T1 -Fi flgK^ were constructed by P22 transduction of the chloramphenicol resistance containing the flgK deletion into the respective host strain. MotA/B were deleted in SL1344 (SB300) using the method of Datsenko and Wanner [63] by insertion of a chloramphenicol resistance cassette that was amplified using the forward primer: ATGCTTATCTTATTAGGTTACCTGGTGGTTATCGGTACAGTGTAGGCTGGAGCTGCTTC and the reverse primer: TCACCTCGGTTCCGCTTTTGGCGATGTGGGTACGCTTGCATGGGAATTAGCCATGGTCC. *S.*Tm^Δ4 motAB^, *S.*Tm^-T1 -motAB^ and *S.*Tm^-T1 -Fi motAB^ were obtained by P22 transduction of the chloramphenicol resistance into the respective host strain. Lack of motility of the respective strains was tested on motility agar.

pM2120 (expressing mCherry constitutively) was constructed by PCR amplification of the mCherry gene using forward primer: CGCGGATCCCCCGGGCTGCAGGAATTCAGGAAACAGTATTCATGGTGAGCAAGGGCGAGGAG (BamHI) and reverse primer: GGGAAGCTTGATATATCGGAATTCTTACTTGTACAGCTCGTCCATG (HindIII). Subsequently the PCR product as well as plasmid pM975 [22] was digested using BamHI/HindIII and ligated.

### List of gene ID numbers of *S*. Typhimurium genes (SL1344)

fliG (11765205), fliH (11765206), fliI (11767468), flgK (11767368), motA (11765169), motB (11765168), cheY (11765165), sopE (11765807), sipA (11765948), sopE2 (11768039), sopB (1252609), invG (11765959), fimD (11764167).

## Supporting Information

Figure S1 **Surface-accumulation of** ***S*** **. Typhimurium requires motility.** It seemed reasonable to assume that two different phenomena might contribute to bacterial accumulation at the bottom of a culture dish, i.e. near surface swimming and gravity. In order to assess the relative importance of gravity, one can compare surface accumulation by motile bacteria (affected by NSS and by gravity) and by non-motile bacteria (affected by gravity, but no by NSS). (A) Experimental design. Glass-bottom dishes were either inoculated with a suspension of motile *S*.Tm^Δ4^ (pGFP)(left panel; green) or with immotile *S*.Tm^Δ4 flgK^(pGFP) (right panel; blue).The number of bacteria present “in solution” (i.e. in the field of view focusing 50 µm, 100 µm and 150 µm above the surface) or “on the surface” was analyzed by confocal time lapse fluorescence microscopy. (B) Glass bottom dishes(w/o cells) were prepared and inoculated with the indicated strains (150 µl of a 4 h sub-culture, 1∶100 diluted in HBSS)exactly as described for Fig. 1. Left panel: To demonstrate a low bacterial density in the 3D volume, images were acquired at the end of the imaging experiment (10 min)in the GFP-channel at Z-slices 50 µm, 100 µm and 150 µm above the surface. The number of bacteria per field of view was plotted. Please note that the chosen density of the bacteria was so low that Z-slices harbored either one (very rare) or no (very often) bacteria. This is true for the *S*.Tm^Δ4^ strain and the *S*.Tm^Δ4flgK^ mutant. Right panel: Confocal time lapse fluorescence microscopy was performed at the Z-slice at the glass surface using a 20× objective with an 1.6 optovar(1 frame per minute; 10 min total; exposure time, 50 ms). The number of bacteria per field of view was quantified in two independent experiments.*S*.Tm^Δ4^ (pGFP) (left panel; green; affected by NSS and by gravity) accumulated at the glass surface (i.e. by NSS) within less than 1 min, while the immotile mutant *S*.Tm^Δ4 flgK^ (pGFP; blue; affected only by gravity) did not. Accumulation of *S*.Tm^Δ4 flgK^ began much later. This demonstrated that gravity had at most a marginal effect in the typical 5 min accumulation experiments performed in our study. Much rather, surface accumulation is driven by NSS.
(EPS)

Figure S2 **Near surface swimming of** ***S*** **. Typhimurium and** ***E. coli*** **Nissle on epithelial cells and on glass surfaces.** A, B. NSS on Madin-Darby canine kidney (MDCK) cells, a commonly used polarized epithelial cell line. MDCK cells were grown to confluence and polarized for 5 days and HeLa cells were grown in glass bottom culture dishes as described in figures 1–3. Wells containing polarized MDCK cells (A, B), HeLa cells (C) or empty wells (D) were infected with *S*.Tm^Δ4^ (pGFP) (A) or *E.coli* Nissle (pGFP) (B–D) as described in figures 1–3. Left panels: DIC image of the respective confluent cell layers. Right panels: Overlay of a 5 min movie in the GFP-channel showing right handed curved bacteria tracks at the cellular surface indicative of bacterial near surface swimming. Scale bar: 25 µm.
(TIF)

Figure S3 **Docking patterns of non-invasive** ***S*** **. Typhimurium mutants with respect to mitotic and non-mitotic cells.** (A)*S*.Tm^Δ4^ (pGFP) docks at the base of rounded cells. HeLa cells were infected with *S*.Tm^Δ4^ (pGFP) for 60 min at an m.o.i. of 62.5, washed, fixed and stained. A 3D-reconstruction of a stack of confocal images is shown. The actin-cytoskeleton is shown in grey, bacteria in green. In addition, extracellular *S*. Typhimurium were stained by an anti-LPS antibody and shown in red. Scale bar: 4 µm. Docking was identical at the 6-min time point (not shown). (B)HeLa cells were infected with the non-invasive strains*S*.Tm^Δ4^(lacks the effectors for triggering membrane ruffles) or *S*.Tm^-T1^(lacks a functional TTSS-1; does not trigger membrane ruffles) for 6 min at the indicated m.o.i. followed by fixation and staining of bacteria, nuclei and actin. *S*. Typhimurium docking onto interphase and mitotic cells was manually quantified using a 40×-objective. The data show a clear target preference for mitotic cells for both mutants. Each bar summarizes 6–7 quantifications from 2 independent experiments summarizing 800–1000 interphase cells or 155–195 mitotic cells, respectively. Technical note: It should be noted that the numbers of bacteria per infected mitotic cell detected in this experiment (*S*.Tm^Δ4^, red bars) was higher than in Fig. 6E (*S*.Tm^Δ4^, dark grey bars). This has technical reasons. In suppl. Fig. S3B we used manual quantification of all individual bacteria from all focus planes of a given infected cell. In contrast, during automated image analysis (in Fig. 6), a single focus layer was quantified. Thus, bacteria located “on top of each other” would be superimposed. Therefore, the detected number of bacteria docked onto mitotic cells is somewhat higher in suppl. Fig. S3 than in Fig. 6. However, this does not affect our conclusions.
(EPS)

Figure S4 ***S*** **. Typhimurium docking is dependent on motility.** HeLa cells were infected with motile and non-motile *S.* Typhimurium mutants for 6 min. Mutants were constructed in the background strains *S*.Tm^Δ4^, *S*.Tm^-T1^and *S*.Tm^-T1-Fi^, as indicated. *S*.Tm^-T1^ is motile, but lacks a functional TTSS-1 apparatus required for efficient docking and the triggering of host cell invasion [9]. *S*.Tm^-T1-Fi^ lacks the TTSS-1 apparatus and the *fim* adhesin which contributes to reversible host-cell binding. These background strains carried additional mutations in *fliGHI* (no flagella), *flgK* (truncated flagella) or *motAB*(do not rotate the flagella), rendering the bacteria non-motile. Docking was analyzed as described [9]. The behavior of all three non-motile mutants on motility agar and in the docking experiment was indistinguishable (data not shown), pointing to a role of motility, not flagella *per se* in *S*. Typhimurium docking. Please note the different scale of the three diagrams which are in line with previous work demonstrating a role of TTSS-1 and the *fim* adhesin in host-cell binding [9]. Data show median and standard deviation from 3 independent experiments.
(EPS)

Figure S5 **Stimulation of** ***S*** **. Typhimurium binding/docking by latrunculin B.** Cells were pre-treated with latrunculin B for one hour prior to infection with the indicated *S.* Typhimurium strain for 6 min. Experimental setup and analysis was identical to the experiment shown in Fig. 7B.
(EPS)

Figure S6 **Automated analysis demonstrates stimulation of** ***S*** **. Typhimurium docking by cellular ruffling.** (A–D) Cells with associated reporter and helper bacteria. HeLa cells were infected for the indicated time at an m.o.i. of 250 with a mixture of reporter and helper bacteria. Reporter strains were *gfp*-labeled and either *S*.Tm^Δ4^(pGFP) (left panels) or *S*.Tm^-T1^(pGFP) (right panels) were used. The helper strain was *rfp*-labeled (using plasmid pM2120));as a helper, we used either bacteria incapable of triggering ruffles(*S*.Tm^Δ4^ or *S*.Tm^-T1^, panels A and B) or bacteria able to trigger ruffling (*S*.Tm^SopE^, panels C and D). After fixation and staining of the nuclei, automated microscopy and automated image analysis was done using the open source program CellProfiler [58] and custom algorithms. Thereby nuclei were identified and the areas of the cells estimated. “Infectious spots” (one or more bacteria of a given color) were identified in the red and the green channel and allocated to the corresponding cell. Therefore, 4 types of cells can be distinguished: i) Cells with only green or ii) Cells with only red *S.* Typhimurium associated, iii) Cells with both, red and green bacteria associated (shown in yellow) and iv) Cells without any associated bacteria. Please note, that with the helper strain *S*.Tm^SopE^ (panels C and D) more red and green *S.* Typhimurium are recruited to the cells. (E, F):Reporter strain associated with the host cell: The fraction of cells with associated reporter bacteria (green bacteria, either *S*.Tm^Δ4^ or *S*.Tm^-T1^) is indicated. This number is calculated by adding the respective green and yellow bars in panels A–D. Please note, that in this plot we cannot distinguish whether the recruited reporter bacterium remains extracellular or intracellular. (G, H) Invasion by the reporter strain: The fraction of cells with invaded reporter bacteria from a parallel experiment is indicated. In this experiment, reporter bacteria (either *S*.Tm^Δ4^ or *S*.Tm^-T1^) were carrying plasmid pM975. This plasmid specifically induces *gfp*-production in intracellular bacteria. Thus, infected cells can be specifically detected. The invasion experiment and automated quantification of cells with intracellular *S*. Typhimurium was done as described [32]; the percentage of cells with invaded reporter bacteria is given. Invasion of the reporter induced by the helper strain is clearly visible. (I, J) Docking of the reporter strain stimulated by the helper strain: The fraction of cells exclusively harboring extracellular bacteria was calculated by subtracting the fraction of cells with intracellular *S.* Typhimurium(panels E, F)from the fraction of cells with associated *S.*Typhimurium (panels G, H). This calculation is a conservative estimation because cells can harbor both, docked and invaded bacteria. The area between both curves (outside bacteria with helper *S*.Tm^Δ4^ and *S*.Tm^SopE^) represents the fraction of cells with reporter bacteria recruited by ruffling in the most conservative estimation. Taken together, this experiment thus demonstrates that ruffling induced by the helper strain stimulates both: docking to the outside of the cell and cellular invasion of the reporter strain. The data shown are representative of two independent experiments. Similar results were obtained after switching of the plasmids for the reporter and helper strains (data not shown).
(EPS)

Figure S7 **Centrifugation enables immotile** ***S*** **. Typhimurium to dock onto HeLa cells but does not restore the preference for cellular ruffles.** HeLa cells were incubated with a 1∶1 mixture of a motile (*S*.Tm^Δ4^) or immotile (*S*.Tm^Δ4 fliGHI^) *gfp*-labelled reporter strain and a ruffle inducing (*S*.Tm^SopE^) or inactive (*S*.Tm^Δ4^) reporter strain at an m.o.i. of 62.5 for 6 minutes. Combinations were (A, C) *S*.Tm^Δ4^ (pGFP)/*S*.Tm^Δ4^ or *S*.Tm^Δ4^ (pGFP)/*S*.Tm^SopE^;(B, D) *S*.Tm^Δ4 fliGHI^ (pGFP)/*S*.Tm^Δ4^ or *S*.Tm^Δ4 fliGHI^ (pGFP)/*S*.Tm^SopE^ as indicated. In (C, D) centrifugation was performed to enable cellular binding of immotile *S*. Typmiurium. Either docking onto whole non-ruffling cells (grey bars) or cellular ruffles (red bars) was quantified, as indicated. The motile strain*S*.Tm^Δ4^ (pGFP) docked onto cells and(with even higher efficiency) onto cellular ruffles. Without centrifugation, immotile *S*.Tm^Δ4 fliGHI^ (pGFP) did not dock onto cells or ruffles. After centrifugation, *S*.Tm^Δ4 fliGHI^ (pGFP) was found associated with cells and ruffles with equal affinity but no preference for ruffles was detected. As an internal control, the helper strain *S*.Tm^SopE^ was also quantified. This strain associated with ruffles with high affinity in all scenarios (red bars with white stripes). Each plot summarizes binding to 21 to 104 cells or ruffles. *: p<0.05. ***: p<0.0001, Mann-Whitney-U test.
(EPS)

Figure S8 ***E.coli*** **Nissle can target to** ***Salmonella*** **-triggered membrane ruffles.** HeLa cells were infected with a 1∶1 mixture of *S*.Tm^wt^ and *E.coli* Nissle (pGFP) at an m.o.i. of 200 for 6 min, fixed and stained for actin (646-phalloidin), nuclei (DAPI) and *S*. Typhimurium (using an anti-LPS antibody). Images were acquired using a Zeiss Axiovert 200 m inverted microscope equipped with an Ultraview confocal head using a 100× objective. *S*.Tm^wt^ is shown in red, *E.coli* Nissle in green, nuclei in grey and actin in blue. Scale bar: 10 µm. It should be noted that overall binding efficiency of *E.coli* Nissle to HeLa was much lower than in the case of *S*.Tm^Δ4^. This might be attributable to the lack of key adhesins, i.e. the TTSS-1. However, all *E.coli* Nissle bacteria which bound to the cell layer were found to be associated with cellular ruffles.
(EPS)

Video S1 ***S*** **. Typhimurium swimming along the surface of HeLa cells.** HeLa cells were infected with *S*.Tm^Δ4^ at an m.o.i. of 0.5and a DIC movie was acquired using a 63× objective at 23 frames per second and is shown in real time. *S*. Typhimurium move in and out of focus following the cellular surface before stopping at a mitotic cell.
(WMV)

Video S2 **Larger magnification of** ***S*** **.Tm^Δ4^NSSat the surface of HeLa cells from the same experiment shown in Video S1.**
(WMV)

Video S3 ***S*** **. Typhimurium NSS on a glass surface with glass bead obstacles.** Gelatine coated glass beads (500 µm diameter) were placed onto a glass-bottom dish filled with HBSS buffer and the swimming behavior of *S*.Tm^wt^(mCherry) was recorded (20 frames per second; 300 frames) by confocal fluorescence microscopy using a 100× objective (oil NA1.4, Zeiss Axio Observer). The overlay of all frames (maximum intensity plot; ImageJ software) is shown in Fig. 4B.
(WMV)

Video S4 **Stopping of** ***S*** **. Typhimurium atcellular ruffles.** HeLa cells were infected with a 1∶1 mixture of *S*.Tm^Δ4^(pGFP) and *S*.Tm^SopE^(pM2112, constitutive *rfp*-plasmid) at an m.o.i. of 5,6 minutes before movie acquisition. A 2.5 minute DIC movie was acquired using a 63× objective and 2-fold optovar, at 23 frames per second and is shown in real time. Images in the fluorescence channel showed that both *S*.Tm^Δ4^ (non-invasive mutant) and *S*.Tm^SopE^ (invasive mutant) were associated with the membrane ruffle (data not shown).
(WMV)

## References

[1] McGhie EJ, Brawn LC, Hume PJ, Humphreys D, Koronakis V (2009). Salmonella takes control: effector-driven manipulation of the host.. Curr Opin Microbiol.

[2] Patel JC, Galan JE (2005). Manipulation of the host actin cytoskeleton by Salmonella–all in the name of entry.. Curr Opin Microbiol.

[3] Schlumberger MC, Hardt WD (2006). Salmonella type III secretion effectors: pulling the host cell's strings.. Curr Opin Microbiol.

[4] Hapfelmeier S, Stecher B, Barthel M, Kremer M, Muller AJ (2005). The Salmonella pathogenicity island (SPI)-2 and SPI-1 type III secretion systems allow Salmonella serovar typhimurium to trigger colitis via MyD88-dependent and MyD88-independent mechanisms.. J Immunol.

[5] Muller AJ, Hoffmann C, Galle M, Van Den Broeke A, Heikenwalder M (2009). The S. Typhimurium effector SopE induces caspase-1 activation in stromal cells to initiate gut inflammation.. Cell Host Microbe.

[6] Muller AJ, Kaiser P, Dittmar KE, Weber TC, Haueter S (2012). Salmonella gut invasion involves TTSS-2-dependent epithelial traversal, basolateral exit, and uptake by epithelium-sampling lamina propria phagocytes.. Cell Host Microbe.

[7] Reis BP, Zhang S, Tsolis RM, Baumler AJ, Adams LG (2003). The attenuated sopB mutant of Salmonella enterica serovar Typhimurium has the same tissue distribution and host chemokine response as the wild type in bovine Peyer's patches.. Vet Microbiol.

[8] Lara-Tejero M, Galan JE (2009). Salmonella enterica serovar typhimurium pathogenicity island 1-encoded type III secretion system translocases mediate intimate attachment to nonphagocytic cells.. Infect Immun.

[9] Misselwitz B, Kreibich SK, Rout S, Stecher B, Periaswamy B (2011). Salmonella enterica serovar Typhimurium binds to HeLa cells via Fim-mediated reversible adhesion and irreversible type three secretion system 1-mediated docking.. Infect Immun.

[10] Finlay BB, Gumbiner B, Falkow S (1988). Penetration of Salmonella through a polarized Madin-Darby canine kidney epithelial cell monolayer.. J Cell Biol.

[11] Josenhans C, Suerbaum S (2002). The role of motility as a virulence factor in bacteria.. Int J Med Microbiol.

[12] Jarrell KF, McBride MJ (2008). The surprisingly diverse ways that prokaryotes move.. Nat Rev Microbiol.

[13] Baker MD, Wolanin PM, Stock JB (2006). Signal transduction in bacterial chemotaxis.. Bioessays.

[14] Porter SL, Wadhams GH, Armitage JP (2011). Signal processing in complex chemotaxis pathways.. Nat Rev Microbiol.

[15] Dibb-Fuller MP, Allen-Vercoe E, Thorns CJ, Woodward MJ (1999). Fimbriae- and flagella-mediated association with and invasion of cultured epithelial cells by Salmonella enteritidis.. Microbiology.

[16] Eichelberg K, Galan JE (2000). The flagellar sigma factor FliA (sigma(28)) regulates the expression of Salmonella genes associated with the centisome 63 type III secretion system.. Infect Immun.

[17] Jones BD, Lee CA, Falkow S (1992). Invasion by Salmonella typhimurium is affected by the direction of flagellar rotation.. Infect Immun.

[18] La Ragione RM, Cooley WA, Velge P, Jepson MA, Woodward MJ (2003). Membrane ruffling and invasion of human and avian cell lines is reduced for aflagellate mutants of Salmonella enterica serotype Enteritidis.. Int J Med Microbiol.

[19] Lockman HA, Curtiss R (1990). Salmonella typhimurium mutants lacking flagella or motility remain virulent in BALB/c mice.. Infect Immun.

[20] McCormick BA, Stocker BA, Laux DC, Cohen PS (1988). Roles of motility, chemotaxis, and penetration through and growth in intestinal mucus in the ability of an avirulent strain of Salmonella typhimurium to colonize the large intestine of streptomycin-treated mice.. Infect Immun.

[21] Schmitt CK, Ikeda JS, Darnell SC, Watson PR, Bispham J (2001). Absence of all components of the flagellar export and synthesis machinery differentially alters virulence of Salmonella enterica serovar Typhimurium in models of typhoid fever, survival in macrophages, tissue culture invasiveness, and calf enterocolitis.. Infect Immun.

[22] Stecher B, Hapfelmeier S, Muller C, Kremer M, Stallmach T (2004). Flagella and chemotaxis are required for efficient induction of Salmonella enterica serovar Typhimurium colitis in streptomycin-pretreated mice.. Infect Immun.

[23] Cummings LA, Wilkerson WD, Bergsbaken T, Cookson BT (2006). In vivo, fliC expression by Salmonella enterica serovar Typhimurium is heterogeneous, regulated by ClpX, and anatomically restricted.. Mol Microbiol.

[24] Stecher B, Barthel M, Schlumberger MC, Haberli L, Rabsch W (2008). Motility allows S. Typhimurium to benefit from the mucosal defence.. Cell Microbiol.

[25] Roy K, Hilliard GM, Hamilton DJ, Luo J, Ostmann MM (2009). Enterotoxigenic Escherichia coli EtpA mediates adhesion between flagella and host cells.. Nature.

[26] Magariyama Y, Sugiyama S, Kudo S (2001). Bacterial swimming speed and rotation rate of bundled flagella.. FEMS Microbiol Lett.

[27] Vigeant MA, Ford RM, Wagner M, Tamm LK (2002). Reversible and irreversible adhesion of motile Escherichia coli cells analyzed by total internal reflection aqueous fluorescence microscopy.. Appl Environ Microbiol.

[28] Lauga E, DiLuzio WR, Whitesides GM, Stone HA (2006). Swimming in circles: motion of bacteria near solid boundaries.. Biophys J.

[29] Hermansson M (1999). The DLVO theory in microbial adhesion.. Colloids and Surfaces.

[30] Schlumberger MC, Muller AJ, Ehrbar K, Winnen B, Duss I (2005). Real-time imaging of type III secretion: Salmonella SipA injection into host cells.. Proc Natl Acad Sci U S A.

[31] Misselwitz B, Strittmatter G, Periaswamy B, Schlumberger MC, Rout S (2010). Enhanced CellClassifier: a multi-class classification tool for microscopy images.. BMC Bioinformatics.

[32] Misselwitz B, Dilling S, Vonaesch P, Sacher R, Snijder B (2011). RNAi screen of Salmonella invasion shows role of COPI in membrane targeting of cholesterol and Cdc42.. Mol Syst Biol.

[33] DiLuzio WR, Turner L, Mayer M, Garstecki P, Weibel DB (2005). Escherichia coli swim on the right-hand side.. Nature.

[34] Li G, Tam LK, Tang JX (2008). Amplified effect of Brownian motion in bacterial near-surface swimming.. Proc Natl Acad Sci U S A.

[35] Hoffmann C, Galle M, Dilling S, Kappeli R, Muller AJ (2010). In macrophages, caspase-1 activation by SopE and the type III secretion system-1 of S. typhimurium can proceed in the absence of flagellin.. PLoS ONE.

[36] Francis CL, Ryan TA, Jones BD, Smith SJ, Falkow S (1993). Ruffles induced by Salmonella and other stimuli direct macropinocytosis of bacteria.. Nature.

[37] Ginocchio C, Pace J, Galan JE (1992). Identification and molecular characterization of a Salmonella typhimurium gene involved in triggering the internalization of salmonellae into cultured epithelial cells.. Proc Natl Acad Sci U S A.

[38] Finlay BB, Falkow S (1990). Salmonella interactions with polarized human intestinal Caco-2 epithelial cells.. J Infect Dis.

[39] Takeuchi A (1967). Electron microscope studies of experimental Salmonella infection. I. Penetration into the intestinal epithelium by Salmonella typhimurium.. Am J Pathol.

[40] Hardt WD, Chen LM, Schuebel KE, Bustelo XR, Galan JE (1998). S. typhimurium encodes an activator of Rho GTPases that induces membrane ruffling and nuclear responses in host cells.. Cell.

[41] Geoghegan M, Andrews JS, Biggs CA, Eboigbodin KE, Elliott DR (2008). The polymer physics and chemistry of microbial cell attachment and adhesion.. Faraday Discuss.

[42] Strevett KA, Chen G (2003). Microbial surface thermodynamics and applications.. Res Microbiol.

[43] Boks NP, Norde W, van der Mei HC, Busscher HJ (2008). Forces involved in bacterial adhesion to hydrophilic and hydrophobic surfaces.. Microbiology.

[44] Hsu R, Ganatos P (1989). The motion of a rigid body in a viscous fluid bounded by a plane wall.. J Fluid Mech.

[45] Kline KA, Falker S, Dahlberg S, Normark S, Henriques-Normark B (2009). Bacterial adhesins in host-microbe interactions.. Cell Host Microbe.

[46] Darwin KH, Miller VL (1999). Molecular basis of the interaction of Salmonella with the intestinal mucosa.. Clin Microbiol Rev.

[47] Hardt WD, Urlaub H, Galan JE (1998). A substrate of the centisome 63 type III protein secretion system of Salmonella typhimurium is encoded by a cryptic bacteriophage.. Proc Natl Acad Sci U S A.

[48] Zhou D, Chen LM, Hernandez L, Shears SB, Galan JE (2001). A Salmonella inositol polyphosphatase acts in conjunction with other bacterial effectors to promote host cell actin cytoskeleton rearrangements and bacterial internalization.. Mol Microbiol.

[49] Finlay BB, Ruschkowski S, Dedhar S (1991). Cytoskeletal rearrangements accompanying salmonella entry into epithelial cells.. J Cell Sci.

[50] Cain RJ, Hayward RD, Koronakis V (2008). Deciphering interplay between Salmonella invasion effectors.. PLoS Pathog.

[51] Robertson JM, McKenzie NH, Duncan M, Allen-Vercoe E, Woodward MJ (2003). Lack of flagella disadvantages Salmonella enterica serovar Enteritidis during the early stages of infection in the rat.. J Med Microbiol.

[52] Loetscher Y, Wieser A, Lengefeld J, Kaiser P, Schubert S (2012). Salmonella transiently reside in luminal neutrophils in the inflamed gut.. PLoS ONE.

[53] Hoiseth SK, Stocker BA (1981). Aromatic-dependent Salmonella typhimurium are non-virulent and effective as live vaccines.. Nature.

[54] Ehrbar K, Friebel A, Miller SI, Hardt WD (2003). Role of the Salmonella pathogenicity island 1 (SPI-1) protein InvB in type III secretion of SopE and SopE2, two Salmonella effector proteins encoded outside of SPI-1.. J Bacteriol.

[55] Kaniga K, Bossio JC, Galan JE (1994). The Salmonella typhimurium invasion genes invF and invG encode homologues of the AraC and PulD family of proteins.. Mol Microbiol.

[56] Stecher B, Denzler R, Maier L, Bernet F, Sanders MJ (2012). Gut inflammation can boost horizontal gene transfer between pathogenic and commensal Enterobacteriaceae.. Proc Natl Acad Sci U S A.

[57] Hapfelmeier S, Muller AJ, Stecher B, Kaiser P, Barthel M (2008). Microbe sampling by mucosal dendritic cells is a discrete, MyD88-independent step in DeltainvG S. Typhimurium colitis.. J Exp Med.

[58] Carpenter AE, Jones TR, Lamprecht MR, Clarke C, Kang IH (2006). CellProfiler: image analysis software for identifying and quantifying cell phenotypes.. Genome Biol.

[59] Olivo-Marin J-C (2002). Extraction of spots in biological images using multiscale products.. Pattern Recognit.

[60] Horvath P, Wild T, Kutay U, Csucs G (2011). Machine learning improves the precision and robustness of high-content screens – using non-linear multi-parametric methods to analyze screening results.. J Biomol Screen.

[61] Sormaz M, Stamm T, Mourad S, Jenny P (2009). Stochastic modeling of light scattering with fluorescence using a Monte Carlo-based multiscale approach.. J Opt Soc Am A Opt Image Sci Vis.

[62] Schmieger H (1972). Phage P22-mutants with increased or decreased transduction abilities.. Mol Gen Genet.

[63] Datsenko KA, Wanner BL (2000). One-step inactivation of chromosomal genes in Escherichia coli K-12 using PCR products.. Proc Natl Acad Sci U S A.

