# Supplementary figures and images for: Near Surface Swimming of Salmonella Typhimurium Explains Target-Site Selection and Cooperative Invasion
Source: PLoS Pathog. 2012 Jul 26;8(7):e1002810. doi: 10.1371/journal.ppat.1002810 (PMC3406100; doi:10.1371/journal.ppat.1002810)

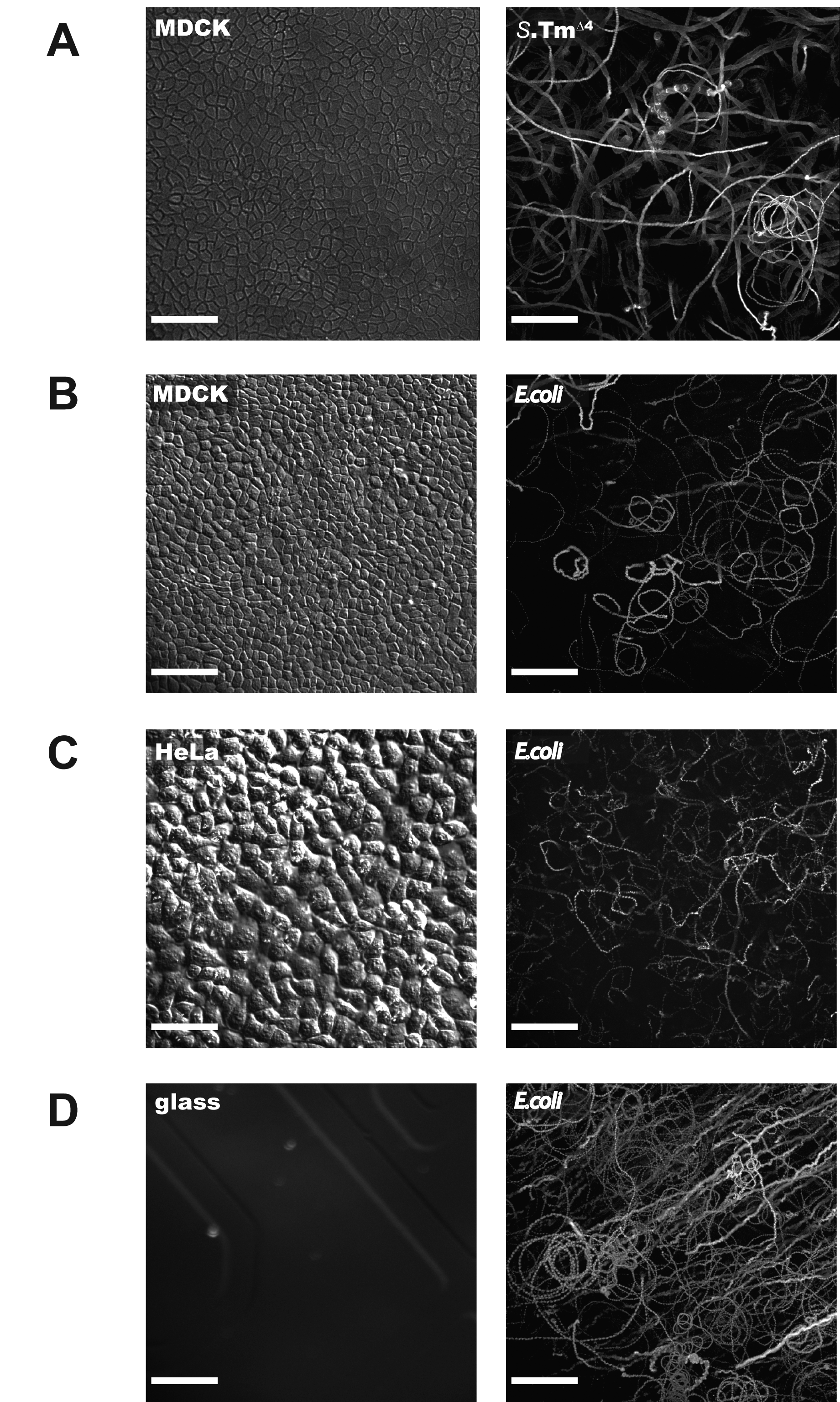

Supplement: Figure S2 — Near surface swimming of S . Typhimurium and E. coli Nissle on epithelial cells and on glass surfaces. A, B. NSS on Madin-Darby canine kidney (MDCK) cells, a commonly used polarized epithelial cell line. MDCK cells were grown to confluence and polarized for 5 days and HeLa cells were grown in glass bottom culture dishes as described in figures 1– 3. Wells containing polarized MDCK cells (A, B), HeLa cells (C) or empty wells (D) were infected with S.TmΔ4 (pGFP) (A) or E.coli Nissle (pGFP) (B–D) as described in figures 1– 3. Left panels: DIC image of the respective confluent cell layers. Right panels: Overlay of a 5 min movie in the GFP-channel showing right handed curved bacteria tracks at the cellular surface indicative of bacterial near surface swimming. Scale bar: 25 µm. (TIF) [file ppat.1002810.s002.tif]
